# Opening the black box: *in situ* imaging of arbuscular mycorrhizal fungal structures in soil using synchrotron‐based micro‐CT

**DOI:** 10.1111/nph.71379

**Published:** 2026-07-02

**Authors:** Henri M. Braunmiller, Michael Bitterlich, Nicolai Koebernick, Eva Jacob, Anna S. Heck, Andrea Schnepf, Johanna Pausch, Jussi‐Petteri Suuronen, Bernhard Hesse, Sylvain Delzon, Jonathan Perrin, Andrew King, Jan Jansa, Mutez A. Ahmed

**Affiliations:** ^1^ Root‐Soil Interaction, School of Life Sciences Technical University of Munich Freising 85354 Germany; ^2^ Division Urban Plant Ecophysiology, Faculty of Life Sciences, Albrecht Daniel Thaer‐Institute for Agricultural and Horticultural Sciences Humboldt‐Universität zu Berlin Berlin 14195 Germany; ^3^ Queensland Alliance for Agriculture and Food Innovation (QAAFI) The University of Queensland, Hermitage Research Facility Warwick QLD 4370 Australia; ^4^ Institute for Bio‐ and Geosciences, Forschungszentrum Jülich GmbH, Agrosphere (IBG‐3) Jülich 52428 Germany; ^5^ Institute of Crop Science and Resource Conservation (INRES) – Soil Science and Soil Ecology University of Bonn Bonn 53115 Germany; ^6^ Agroecology, Bayreuth Center of Ecology and Environmental Research (BayCEER) University of Bayreuth Bayreuth 95447 Germany; ^7^ Xploraytion GmbH Invalidenstraße 34 Berlin 10115 Germany; ^8^ INRAE, Université de Bordeaux UMR BIOGECO Pessac 33615 France; ^9^ Synchrotron SOLEIL L′Orme des Merisiers Saint‐Aubin 91190 France; ^10^ Institute of Microbiology Czech Academy of Sciences Praha 142 00 Czechia

**Keywords:** arbuscular mycorrhizal fungi, functional‐structural soil–plant models, hyphal networks, image‐based modeling, noninvasive imaging, soil texture, X‐ray computed tomography

## Abstract

- Arbuscular mycorrhizal fungi (AMF) contribute to plant nutrient and water uptake via their extraradical hyphal networks. However, *in situ* methodologies to quantify architectural and morphological traits of these networks in soil are largely lacking, limiting our understanding of AMF‐mediated resource transport.
- Using synchrotron‐based X‐ray computed microtomography (micro‐CT), we established a workflow to cultivate, noninvasively image, and quantitatively analyze AMF hyphosphere and rhizosphere structures in the interaggregate space across two soil textures and biological contexts.
- We developed a pipeline for quantitative three‐dimensional (3D) assessment of key architectural and morphological traits including structure counts, hyphal length, branching frequency, volume, and surface area. Our method further permits (1) measurement of AMF–soil and AMF–root interface areas and (2) microscale quantification of pore space occupancy by AMF.
- Micro‐CT offers a tool for noninvasively visualizing AMF in air‐filled soil pore space. We outline how such quantitative 3D information can be incorporated into image‐based and functional‐structural soil–plant models, thereby supporting a better mechanistic understanding of AMF‐mediated processes in soils and plants.

## Introduction

Arbuscular mycorrhizal fungi (AMF) may enhance host plant access to soil nutrients and hence contribute to global food security and nutrition (Antunes *et al*., 2012; Rodriguez & Sanders, 2015; Thirkell *et al*., 2020). These fungi can supply noticeable proportions of essential plant nutrients, including phosphorus, nitrogen, zinc, and copper (Marschner & Dell, 1994). In addition, numerous studies showed that AMF improve plant water uptake, particularly under dry soil conditions (e.g. Bitterlich *et al*., 2018; Kakouridis *et al*., 2022; Abdalla *et al*., 2023), suggesting a potentially increasing contribution to plant performance under future climate scenarios. A proposed mechanism for AMF‐mediated enhancement of water and nutrient acquisition involves the formation of extensive extraradical hyphal networks (Kokkoris, 2026). These networks increase the absorptive surface area and enlarge the effective root radius (Abdalla & Ahmed, 2021), growing beyond depletion zones and thus facilitating access to yet undepleted soil (Schnepf *et al*., 2008). The degree to which AMF improve plant water and nutrient uptake likely depends on the architecture and morphology of their hyphal network, including hyphal length, diameter, and connectivity among individual hyphae, roots, and soil particles (Abdalla & Ahmed, 2021; Kakouridis *et al*., 2022). Thus, for a better mechanistic understanding of the role of AMF on water and nutrient transport, characterization of hyphal network structure in soil is indispensable.

### Undisturbed AMF imaging in transparent Petri dish setups

To date, most information on undisturbed AMF growth patterns has been obtained in highly controlled, artificial setups. A substantial portion of this research relies on only a few AMF species, predominantly *Rhizophagus irregularis* and *Gigaspora margarita*, often grown in soilless Petri dish‐based root‐organ cultures. Such monoxenic cultures, established decades ago (Mosse & Hepper, 1975; Bécard & Fortin, 1988), have been instrumental in elucidating how AMF and their extraradical mycelium respond to specific environmental factors. Their major advantage lies in the user's ability to manipulate and control environmental conditions precisely, while the transparency of the setup enables high‐resolution, nondestructive imaging. Early work used light microscopy to characterize fundamental aspects of AMF extraradical hyphal behavior, including tip growth, branching, and anastomoses (Bécard & Fortin, 1988; Chabot *et al*., 1992, Bago *et al*., 1998; Giovannetti *et al*., 1999; De La Providencia *et al*., 2005). Subsequently, fluorescent markers greatly improved visualization of intraradical AMF structural development (Vierheilig *et al*., 2001; Aguilera *et al*., 2011; Floss *et al*., 2013). Other methods enabled visualization of cytoplasmic streaming within extraradical hyphae (Bago *et al*., 2002; Whiteside *et al*., 2019; Van't Padje *et al*., 2021). Imaging setups were also complemented by semi‐automated image analysis pipelines, which accelerated the quantification of hyphal network architecture and growth trajectories (Fricker *et al*., 2008; Cardini *et al*., 2020; Dikec *et al*., 2020; Martinez‐Galicia *et al*., 2023). The analytical innovations set the stage for recent automated imaging approaches that resolve AMF hyphal development both at high temporal and spatial resolution. For instance, Oyarte Galvez *et al*. (2025) have used such approaches to demonstrate that hyphal growth, branching, fusion, and cytoplasmic flow can be tracked across entire mycelial networks. This showed mechanistic links between architectural and morphological development as well as resource allocation dynamics. Taken together, imaging in monoxenic Petri dish systems has enabled the coupling of AMF architectural traits with metabolic processes under controlled‐environmental conditions. However, in natural environments, AMF experience substantial heterogeneity of soil, and their growth patterns and resource allocation strategies may differ substantially from those observed in simplified *in vitro* settings.

### Nondestructive AMF imaging in obstacle chips

To bridge the gap between controlled *in vitro* systems with homogeneous media and the physical complexity of soil, researchers have utilized soil chips (Hanson *et al*., 2006; Stanley *et al*., 2016; Mafla‐Endara *et al*., 2021). These setups provide a transparent, flat growth environment with a predefined geometric pore network, facilitating high‐resolution observation of soil microorganisms. Using this approach, Aleklett *et al*. (2021) and Hammer *et al*. (2024) investigated the response of fungal growth to variation in pore geometry in both saprotrophic fungi and AMF. Their findings revealed species‐specific responses to pore shape and different hyphal exploration strategies. Among seven saprotrophic fungal species, Aleklett *et al*. (2021) documented a range of branching behaviors, growth rates, extension distances, and length densities. Hammer *et al*. (2024) applied a similar soil chip‐framework to AMF (*R. irregularis*) to determine whether pore geometry influenced the growth patterns. The authors found a pronounced increase in branching frequency – from *c*. 8 to 95% – in response to physical obstacles. Moreover, channel shape strongly affected hyphal growth: Bends > 90° markedly reduced the maximum distance hyphae were able to traverse. Similarly, Richter *et al*. (2024) grew different AMF species, *R. irregularis*, *R. intraradices*, and *G. margarita*, from spores on a transparent chip and studied germination, hyphal emergence, and anastomosis events. The freshly germinated fungi were subjected to different obstacles and showed branching, septa formation, and cytoplasmic retraction as a response to hyphal entrapment. Together, these studies demonstrated that pore geometry affects mycelial development, implying that soil structure shapes AMF hyphal architecture and morphology. Still, such soil chips represent only a simplified approximation and do not reflect the true three‐dimensional (3D) physical and chemical complexity of soil. Since hyphal exploration is strongly influenced by resource gradients and their spatial distribution, assessing AMF architecture and morphology in soil is a necessity.

### Destructive and nondestructive quantification of AMF architecture in soil

A wide range of methods have been used to study AMF architecture and morphology in soil. Using destructive harvests, Jakobsen *et al*. (1992) quantified temporal changes in extraradical hyphal length density at increasing distances from roots, showing strong species‐specific differences: Some species produced high densities near roots that declined rapidly with distance, whereas others maintained lower but more consistent densities at up to 10‐cm distance. Similarly, Drew *et al*. (2003) demonstrated species‐specific differences in hyphal length and diameter, with soil texture exerting a strong influence, although only sandy textures were examined. Early nondestructive approaches employed transparent observation windows in tilted root chambers to qualitatively assess AMF hyphal architecture directly in soil (Friese & Allen, 1991), and to quantify hyphal diameters by branching order. Combining root chambers with destructive hyphal extraction, Hart & Reader (2005) quantified infection units, germinating hyphae, root contact points, hyphal bridges, and the relative abundance of runner vs absorptive hyphae. Weekly measurements revealed clear temporal and species‐specific differences across three AMF species. In a complementary approach, root systems were constrained to a flat plane between meshes in sterile sand and inoculated with AMF (Giovannetti *et al*., 1993, 2001). Excavation of intact root systems enabled visualization and quantification of complete extraradical hyphal networks, including total hyphal length, surface coverage, and anastomosis frequency. This whole‐root perspective further allowed comparisons between hyphal and root lengths, revealing plant host‐dependent hyphae‐to‐root length ratios ranging from 22 : 1 to 74 : 1 (Giovannetti *et al*., 2001). More recently, the ‘AMF slide’ developed by McGaley *et al*. (2025) revived gravity‐based root positioning at a transparent plane, enabling nondestructive live imaging of colonized roots and *in situ* confocal imaging of arbuscule development. Despite the insights provided by these nondestructive systems, they all share key limitations: Growth and observation are largely constrained to two dimensions (2D), *z*‐axis depth is limited, and particularly for extraradical hyphae, overlap with soil complicates observation and thus can generate artifacts, and true 3D parameters such as volume and surface area are difficult to quantify.

### Noninvasive imaging of AMF in soils using micro‐CT

The opaque nature of soil impedes direct observation of roots and their associated fungi demanding alternatives to visible light microscopy. Synchrotron radiation‐based X‐ray computed microtomography (micro‐CT) is a technique that has been successfully applied to capture microscopic root features, including root hairs (Koebernick *et al*., 2017; Duddek *et al*., 2022), and more recently, AMF hyphae in soil (Keyes *et al*., 2022). Although laboratory‐based CT systems show considerable potential for detecting AMF structures (Duncan *et al*., 2022, 2023), it is the high brilliance of synchrotron radiation sources, which offers a major advantage by significantly accelerating scan times, making sample stability less of a concern and allowing high throughput. Using a pot setup that contained root‐exclusion compartments, Keyes *et al*. (2022) used micro‐CT to image fungal hyphae in a loam‐textured soil and analyzed architectural traits including hyphal length density, branching frequency and angles, and tortuosity. A key limitation is that only hyphae growing in air‐filled pores have sufficient contrast and can thus be detected. In most experimental setups, complete drainage of water would be harmful to the observed organisms and water in micropores must remain. This means that contrast of hyphae growing in micropores would be obscured by water. The limitation also remains in this study.

Despite this, micro‐CT currently represents the most advanced method for noninvasively capturing the 3D architecture of pore‐bridging hyphae within intact soil pore networks. Its *in situ*, 3D imaging capability allows for direct linkage of extra‐ and intraradical AMF structures to surrounding soil structural properties and supports subsequent destructive analyses on the same samples. Importantly, micro‐CT provides access to architectural parameters previously inaccessible, including 3D growth angles, branching behavior relative to roots, and interface areas between AMF structures, soil, air, and roots. These are key traits for understanding fungal‐mediated water and nutrient transport in soils.

### Objectives and approaches

We used synchrotron‐based micro‐CT to characterize undisturbed AMF architecture and morphology, thereby improving our understanding of the mechanisms behind AMF‐mediated water and nutrient transport. With micro‐CT, we aimed to visualize how fungal hyphae utilize the soil pore space and to observe their behavior near mineral soil particles and roots *in situ*. Our specific objectives were (1) to develop simple setups that produce easily extractable, representative subvolumes that allow for micro‐CT imaging and contain AMF structures in different soil textures and biological contexts; (2) to successfully carry out micro‐CT imaging of the AMF structures; and (3) to establish a segmentation and image analysis pipeline that allows for the extraction of architectural and morphological features *in situ*. To accomplish the first goal, we used *R. irregularis* in a monoxenic Petri dish culture with Ri‐T‐DNA‐transformed chicory (*Cichorium intybus* L.) roots as the host (Rozmoš *et al*., 2022). Additionally, we cultivated *Solanum lycopersicum* L. colonized by one of two AMF species, namely *R. irregularis* and *Funneliformis mosseae*, in two contrasting soil textures, namely a coarse sand and a silty loam. Both setups contained ingrowth cores (representative subvolumes) with intact soil structure that could be subsequently imaged with synchrotron‐based micro‐CT and semi‐automatically segmented and analyzed with image analysis tools. In short, in this method paper, we describe our methodological procedures and discuss their limitations and potential improvements. Finally, we outline how image‐based and functional‐structural soil–plant models may utilize such data.

## Materials and Methods

**Fig. 1:**
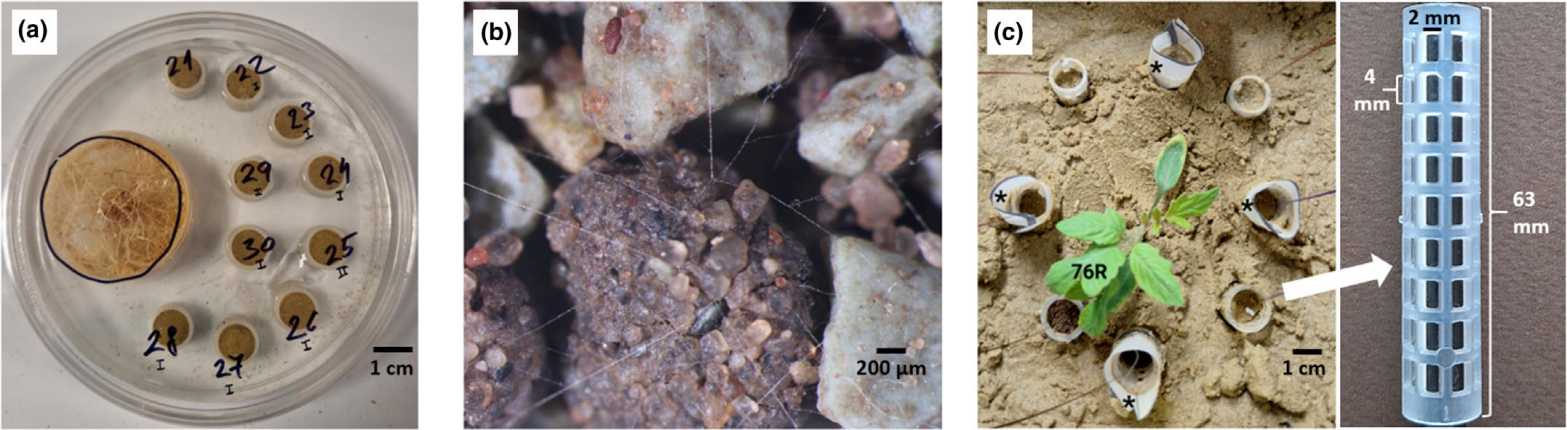
Experimental setups. (a) *Sterile Hyphosphere* setup with Ri‐T‐DNA–transformed chicory roots (*Cichorium intybus* L.) confined to a root compartment (circled, left) and soil‐filled ingrowth cores (numbered) used for computed microtomography imaging. (b) Extraradical hyphae of an arbuscular mycorrhizal fungus (*Rhizophagus irregularis*) spanning between soil particles in the *Sterile Hyphosphere*, imaged with light microscopy. (c) *Hyphosphere* and *Rhizosphere* pot setup with *Solanum lycopersicum* L. (cv 76R) surrounded by ingrowth cores either wrapped with a nylon mesh (*; *Hyphosphere*) or without mesh (*Rhizosphere*, see on the right).

We designed two complementary model systems for the noninvasive visualization of AMF in soils. The first is a monoxenic Petri dish–based microcosm that includes a host plant root system, the arbuscular mycorrhizal fungus, and soil‐filled ingrowth cores (Fig. 1a,b). We call this system the ‘*Sterile Hyphosphere*’, as the ingrowth cores contain a soil zone that is only accessible to AMF. The second system consists of a pot setup equipped with ingrowth cores (Fig. 1c) that contain either (1) only extraradical AMF structures (‘*Hyphosphere*’) or (2) both roots and AMF structures (‘*Rhizosphere*’).

### Setup *Sterile Hyphosphere* for imaging AMF structures in sterile conditions

The primary objective of the *Sterile Hyphosphere* setup was to generate samples containing exclusively AMF structures. The setup (*n* = 6) consisted of Ri‐T‐DNA‐transformed chicory (*Cichorium intybus* L.) ‘immortal’ roots, which served as a continuous carbon source for *R. irregularis* (isolate LPA9 = BEG 236). The chicory roots and the fungus were cultivated in the dark at room temperature for 3.5 months.

During cultivation, the fungus extended its extraradical hyphae beyond the root compartment through a 42‐μm root‐exclusion mesh and colonized the surrounding growth medium, which was solidified with 0.4% Phytagel (c.f. Rozmoš *et al*., 2022). The medium was autoclaved and the root compartments were gamma‐irradiated (> 25 kGy) before use. The soil‐filled ingrowth cores were packed using previously dried soil and gamma‐irradiated (> 25 kGy) before placing them into the medium at a distance of 2–5 cm from the root compartment (Fig. 1a). After 6 wk, when the AMF started forming extraradical hyphae, the soil‐filled ingrowth cores were placed into the gel (Fig. 1a,b). Such subvolumes are needed for micro‐CT, as soil mineral particles strongly attenuate X‐rays at the typical photon energies at synchrotron micro‐CT beamlines (*c*. 20–50 keV). Thus, sample size is limited to a maximum diameter of *c*. 1 cm retaining enough transmission for micro‐CT imaging. The ingrowth cores contained a silty loamy soil (Supporting Information Table S1). Each core was sealed on all sides except the top, in which hyphae were allowed to enter. The soil‐filled ingrowth cores could be removed easily and intact for scanning.

### Setups *Hyphosphere* and *Rhizosphere*: imaging AMF structures in unsterile soil

Tomato plants (*Solanum lycopersicum* L., genotype 76R, *n* = 24) were grown for 8 wk in a controlled‐environment chamber (relative humidity 65%; day: 14 h, 24°C, 400 μmol m^−2^ s^−1^ light; night: 10 h, 22°C, dark). Three times a week, the pots were watered to 80% of water‐holding capacity. Each pot contained 3 l of one of two initially autoclaved, low‐phosphorus, organic‐poor soils: a fine sand or a silty loam with respective bulk densities of 1.50 and 1.22 g cm^−1^ (Table S1). Two AMF species were used as inocula. Half of the pots were inoculated with 5% (v/v) *R. irregularis* (BEG 158) and the other half with *F. mosseae* (BEG 161), homogeneously mixed with the autoclaved soil. The inoculum consisted of AMF spores, hyphae, and colonized root fragments as well as an accompanying soil microbiome. It was generated by propagating leek‐derived AMF pot cultures in sterilized silty loam with maize (*Zea mays* L.) for 3 months, using 5% (v/v) substrate transfer to avoid strong changes in soil texture. These two fungal species were selected because of their contrasting extraradical hyphal architectures and divergent responses to soil texture (cf. Drew *et al*., 2003).

The tomato plants were surrounded by multiple cylindrical ingrowth cores (Fig. 1c; diameter 12 mm, height 60 mm, sides are perforated with 4 × 2 mm windows), which contained either (1) pure coarse sand or (2) silty loam (soil properties provided in Table S1). Both soils were sterilized and contained no AMF inoculum. The ingrowth cores were placed 3 cm away from the seedling, and during installation, we tried to avoid any soil compaction. A subset of cores was wrapped in a 37‐μm nylon mesh (with an open area of 24% and a thickness of 60 μm; Franz Eckert GmbH, Waldkirch, Germany) to prevent root ingrowth while allowing hyphal ingrowth. These mesh‐wrapped cores are further referred to as the *Hyphosphere*. The remaining cores were left unwrapped to permit root ingrowth and thus enabled imaging of *Rhizosphere* structures, including both extra‐ and intraradical AMF features.

### Synchrotron‐based micro‐CT imaging

Watering of the pots was stopped 2 d before micro‐CT imaging to drain macro‐ and mesopores. This step improved hyphal visibility by reducing water content, as fungal hyphae have X‐ray attenuation values similar to those of water. Consequently, the soils were at or below field capacity while pores < 30 μm were potentially still water‐filled. At the beamline, the ingrowth cores were carefully removed from both the Petri dishes and pots. In the pots, the soil around the ingrowth cores was removed, and all connecting roots were cut. Each ingrowth core sample was removed only shortly before imaging and wrapped in a wax film (PARAFILM M, Heathrow Scientific, IL, USA) to minimize desiccation and prevent soil disturbance during handling. For the *Sterile Hyphosphere* samples, micro‐CT scans were acquired near the top of the ingrowth cores in which hyphal ingrowth had been confirmed microscopically. For the *Hyphosphere* and *Rhizosphere* samples, scans were conducted at the center of the ingrowth cores (h = 3–4 cm), the positions in which we expected the least disturbance during their removal from the setup and decent hyphal density.

Synchrotron‐based micro‐CT imaging was performed at the PSICHÉ and ANATOMIX beamlines at Synchrotron SOLEIL, St. Aubin, France. At both beamlines, overview scans with a resolution of 2.8 μm (PSICHÉ) or 3 μm (ANATOMIX) per voxel and a total field of view (FOV) of *c*. 11 × 11 × 5.5 / 12 × 12 × 12 mm were performed. This was done to select regions containing AMF structures for subsequent high‐resolution scans (at 0.65 μm voxel size, FOV of *c*. 1.5 × 1.5 × 1.5 mm at PSICHÉ and 3.0 × 3.0 × 1.5 mm at ANATOMIX). The detection limit for structures was *c*. 2 μm (3 voxels). For more details on beamline‐specific settings, we refer to the Notes S1.

### Image segmentation

**Fig. 2:**
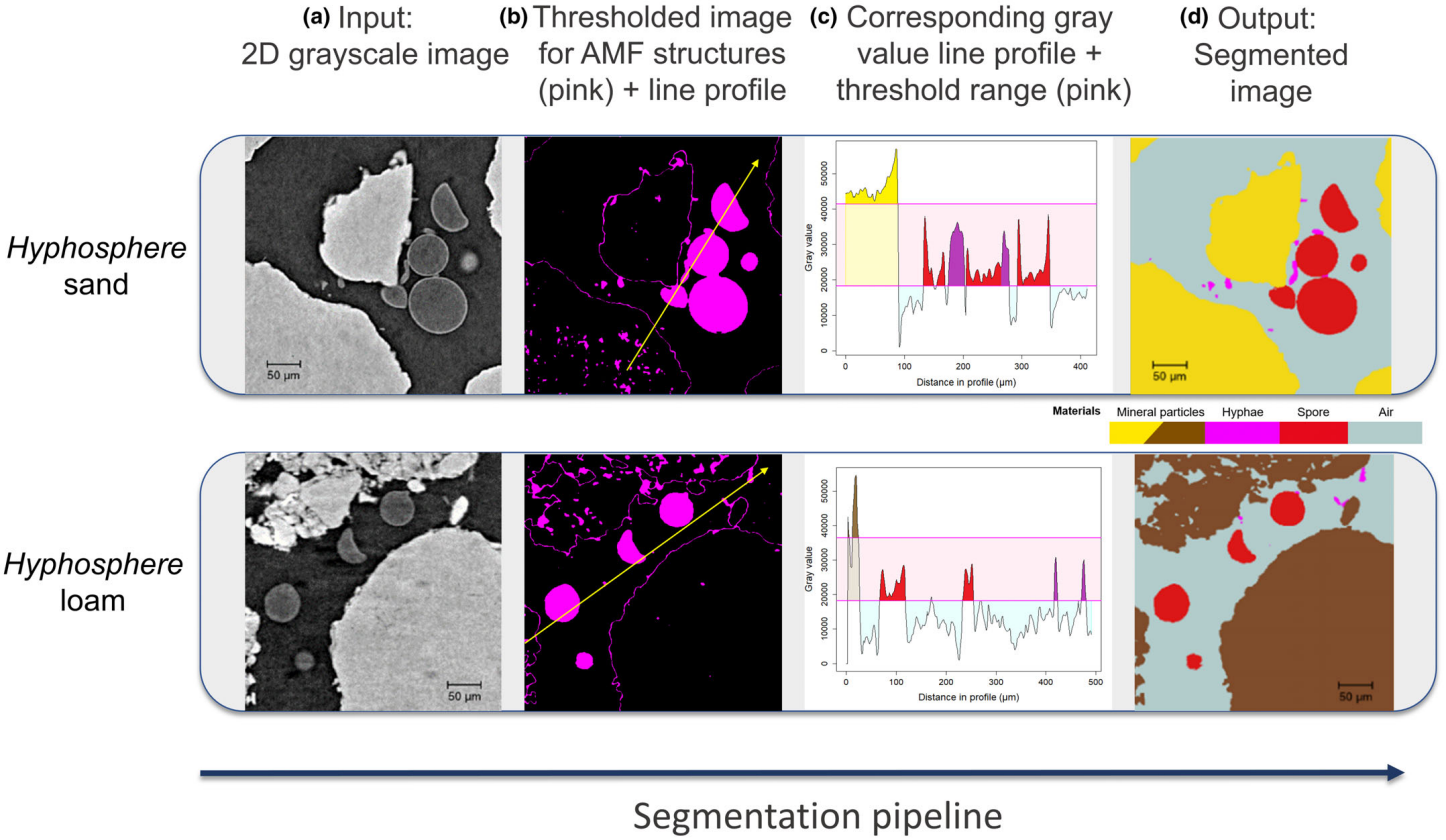
Segmentation workflow from grayscale input to classified image. Example images illustrate the main image processing steps for hyphae and spores of an arbuscular mycorrhizal fungus (species: *Rhizophagus irregularis*) in the *Hyphosphere* setup for coarse sand and silty loam. (a) The grayscale input is noise‐filtered and (b) thresholded to extract intermediate‐density structures (in pink color). The yellow transect in (b) corresponds to the gray value line profile (c). The pink region in (c) signifies the range between the two thresholds used to extract the intermediate‐density structures in (b). The structures are then assigned to different material classes (d). A more detailed overview of the steps is provided in the Supporting Information Fig. S1.

Image preprocessing and segmentation were performed using ImageJ (Schindelin *et al*., 2012) and Avizo (Thermo Fisher Scientific, Waltham, MA, USA). The segmentation workflow consisted of several steps. First, a 3D median filter (two iterations, 26‐pixel neighborhood) was applied to reduce image noise. The air‐filled pore space was then segmented via manual thresholding, leaving solid particles and water‐filled pores in the foreground. A second manual threshold was used on the foreground to isolate intermediate‐density features (roots, AMF structures, and water) from the higher density mineral phase (Fig. 2). To correct for local misclassifications and partial‐volume artifacts, we applied two‐dimensional flood‐fill and erosion operations to the intermediate‐density class (Fig. S1). Subsequently, fungal hyphae and spores were manually classified in Avizo using the ‘Segmentation Editor’, which enabled assignment of these features to separate ‘hyphae’ and ‘spore’ classes in the *Hyphosphere* volumes (Fig. 2). The features could only be segmented in meso‐ and macropores, as water was remaining in the micropores. An analogous classification procedure was used for roots, root hairs, and intraradical AMF structures. In the *Rhizosphere* volumes, we distinguished turgid root hairs from thinner filaments (shrunken root hairs or hyphae) based on diameter and morphology (Notes S2). Previous work (Duddek *et al*., 2022, 2023) has shown that root hairs can undergo localized shrinkage even at modest matric potentials (between −10 and − 310 kPa). Reliably differentiating shrunken root hairs from hyphae can be challenging, although hyphal branching remains a diagnostic feature. To accommodate this uncertainty, we grouped these filaments into a unified class of ‘thin filaments’ and subsequently compared their morphology with that of clearly identifiable turgid root hairs and clear hyphae from the *Hyphosphere* volumes.

### Image analysis

Detailed image analyses were carried out on two segmented volumes from *Hyphosphere* samples (root exclusion; 975 × 390 × 390 μm) as well as four segmented volumes from *Rhizosphere* samples (195 × 195 × 195 μm). The latter four volumes consisted of two contrasting soil textures, sand and loam, and two AMF species. Segmented images were processed and analyzed in ImageJ (Schindelin *et al*., 2012). Before quantitative analysis, each segmented phase was smoothed with a 3D median filter (kernel length: 2 voxels). On the resulting binary images, volume and surface area were quantified using the ‘3D Analyze’ tool from the MorphoLibJ plugin (Legland *et al*., 2016). Interfacial surface areas between pairs of phases were obtained using the ‘Interface Surface Area’ tool in MorphoLibJ; only structures larger than 100 μm^3^ were included in this analysis.

**Fig. 3:**
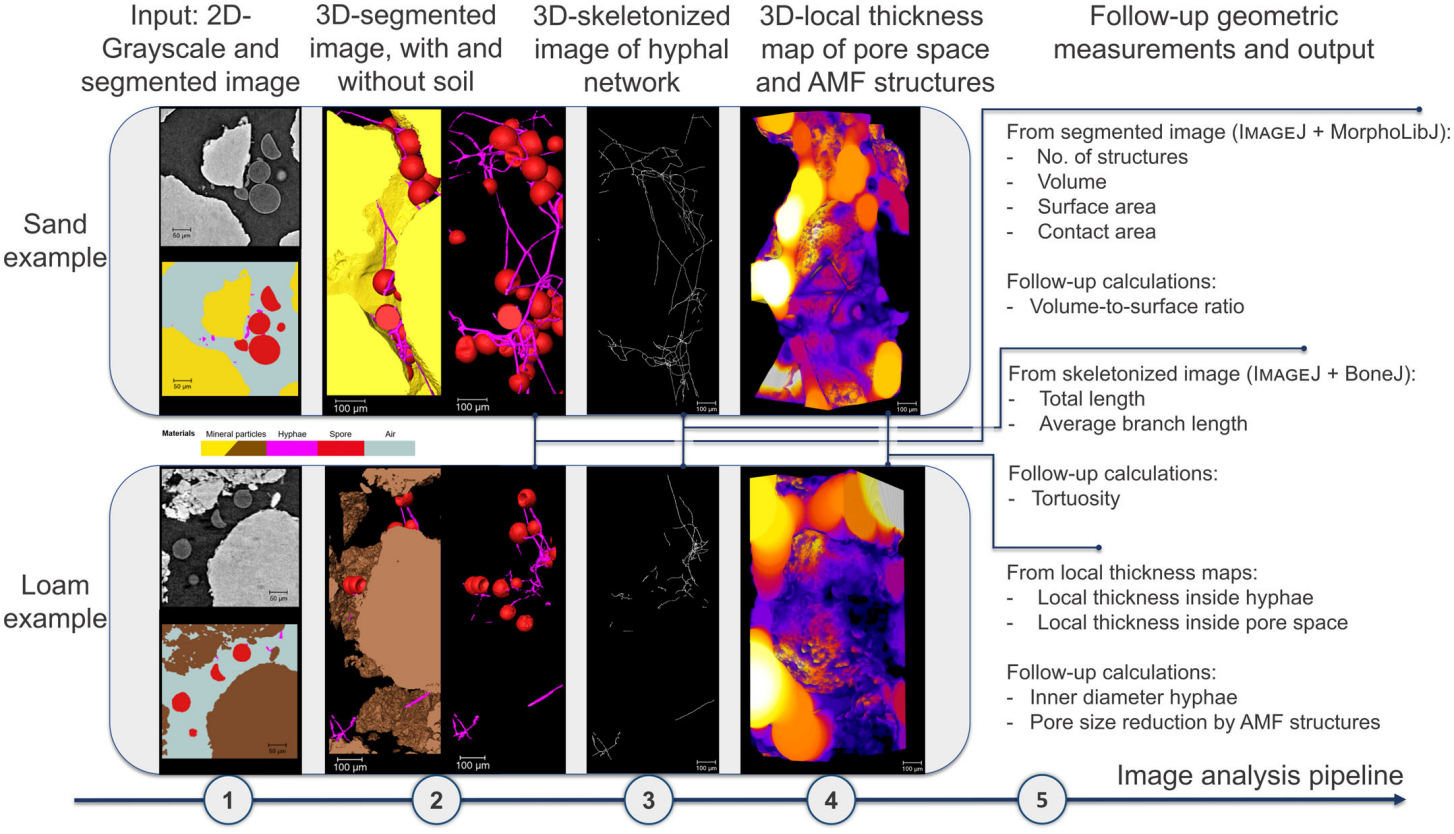
Three‐dimensional (3D) image analysis pipeline. Exemplary *Hyphosphere* samples containing an arbuscular mycorrhizal fungal (AMF, species: *Rhizophagus irregularis*) spore cluster and associated hyphae, in sand and loam respectively, illustrate the processing steps. (1) 2D grayscale image and its corresponding segmented image (390 × 390 μm). (2) 3D segmented volume (975 × 390 × 390 μm) along with the same volume rendered without soil to visualize only the hyphal network and spores. (3) 3D skeleton representation of the segmented hyphal network. (4) 3D local‐thickness map of the soil pore space in the presence of AMF. (5) Derived geometric parameters generated from segmented and post‐processed images.

Hyphae and root hair phases were skeletonized and analyzed with BoneJ's ‘Analyze Skeleton’ tool (Domander *et al*., 2021; Fig. 3) to derive segment length and tortuosity. The output skeletal branch length should not be confused with biological branching. From the images, it was not clear whether the hyphae really branched or simply those junctions represented several hyphae in close contact without cytoplasmic continuity. Only skeletal branches longer than 26 μm were evaluated to exclude discontinuous segments that were below our resolution limits. Tortuosity was defined as the ratio of segment length to the Euclidean distance between its endpoints. Hyphal diameter was determined using BoneJ's ‘Local Thickness’ tool, and intersecting the resulting thickness map with the skeleton provided diameter values along the hyphal length. Gray value distributions of distinct materials were quantified by multiplying each binary mask with the original grayscale image and analyzing the resulting histograms. The impact of AMF structure presence on pore size dimensions was quantified by calculating local thickness of the air‐filled pore space before and after virtual removal of all AMF structures (i.e. adding them to the pore phase). This enabled estimation of the reduction in average pore size attributable to the presence of AMF and thus the pore space occupancy (Fig. 3). Details of the complete workflow are provided in Notes S3. For measurement of the length and width of intraradical structures, the ‘measure line’ tool in Avizo was used.

### Quantification and visualization

Colonization assessments (Table S2) and geometric measurements from the exemplary image analyses were averaged across replicates. For comparing geometric features of AMF hyphae‐like structures (thin filaments) with those of turgid root hairs, the example volumes were pooled. Visualization of 2D grayscale images and maximum intensity projections was performed in ImageJ (Schindelin *et al*., 2012). Maximum intensity projections display for each pixel the maximum gray value detected along the projection axis. Avizo was additionally used for visualization of 2D grayscale images, segmented images, and 3D renderings. Line graphs, violin, and box plots were generated in R v. 4.3.2 (R Core Team, 2023).

## Results

For the *Sterile Hyphosphere* setup, optical stereomicroscopy qualitatively confirmed that *R. irregularis* hyphae and spore density were high in the Phytagel medium. Hyphae crossed the rim of the ingrowth cores and proliferated across the soil surface (Fig. 1b). For the *Hyphosphere* and *Rhizosphere* setups, AMF root colonization frequency ranged between 85% and 100% of total root length for both fungal species. In *Hyphosphere* and *Rhizosphere* ingrowth cores, we detected 28S rDNA copies of the inoculated AMF species (Table S2). Because the soil in the ingrowth cores was sterilized and received no inoculum, the presence of DNA provides evidence that the freshly ingrown AMF species were present. The hyphal length density in the *Rhizosphere* ingrowth cores ranged from 0.10 to 2.24 m g^−1^ soil. For more details, we refer to the Tables S2 and S3.

### Imaging of AMF extraradical structures in the *Sterile Hyphosphere* setup

**Fig. 4:**
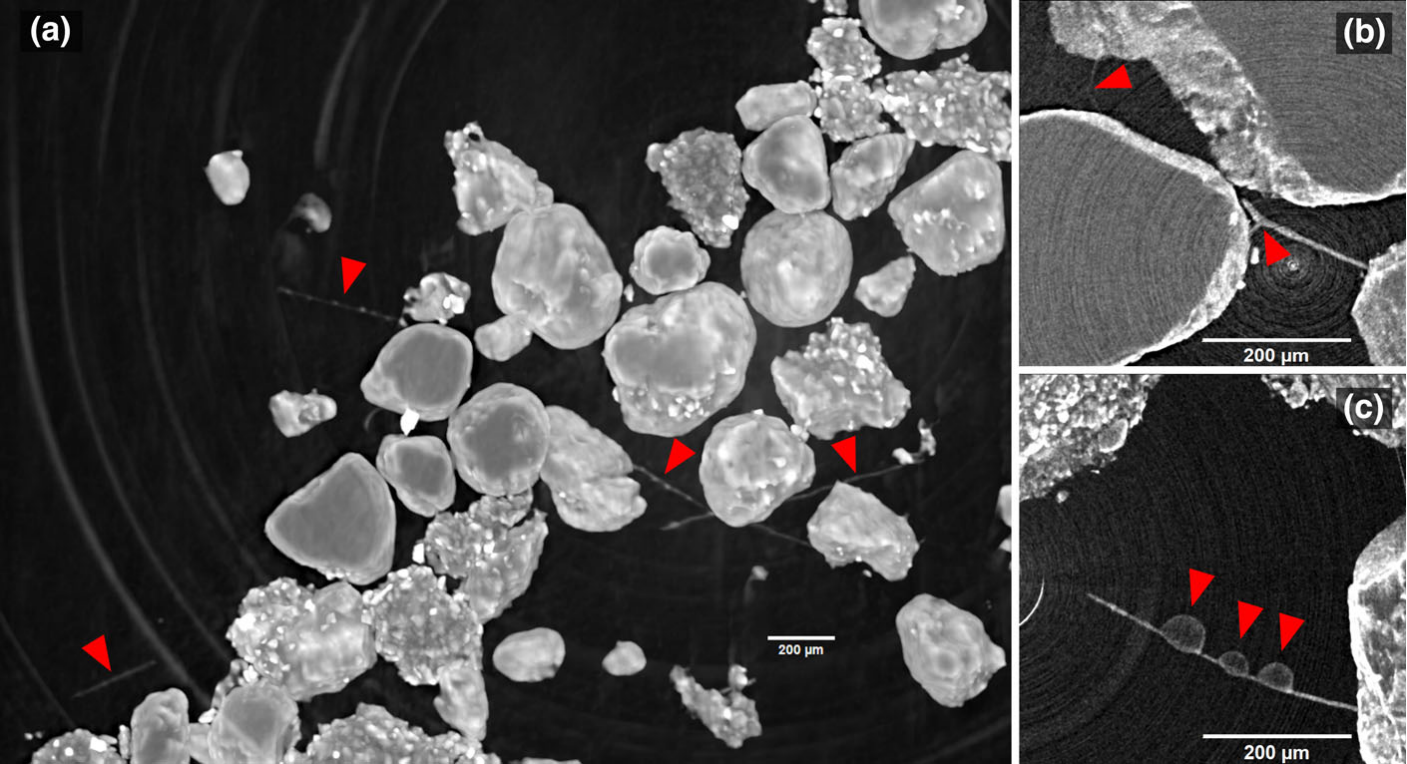
Maximum intensity projections of computed microtomography images of arbuscular mycorrhizal fungal (AMF, species: *Rhizophagus irregularis*) hyphae (red arrows) in the *Sterile Hyphosphere* setup. (a) Overview scan (2.8 μm voxel size, field of view: 4.5 × 2.8 mm) of the top 120 μm of the soil core, showing multiple hyphae spanning pores. (b, c) High‐resolution scans (0.65 μm voxel size, field of view: 0.5 × 0.5 mm; height: 120 μm). (b) Two branching AMF hyphae (branch points indicated by arrows). (c) Three droplets along a hypha.

Micro‐CT scans demonstrated that hyphae were present within air‐filled pores in both overview scans (2.8 μm voxel size) and high‐resolution scans (0.65 μm voxel size). Relative to air, their absorption and phase‐contrast properties yielded distinct gray value peaks. In overview scans, hyphae were detectable but not fully resolved as continuous filaments; rather, they appeared as discontinuous segments. Nevertheless, we observed *R. irregularis* hyphal branching and spanning the pore space between soil particles (Fig. 4a). High‐resolution scans captured individual hyphae as mostly continuous filaments that occasionally branched (Fig. 4b). Droplets frequently formed along hyphae located in pores wider than *c*. 100 μm (Fig. 4c). Across three scans covering the top 1.2 mm of the ingrowth core (surface area: 1.4 mm^2^), we identified a total of 12 discrete hyphae. The deepest hypha detected in high‐resolution scans was located at 0.9 mm below the surface. In three additional scans acquired at 1–3 mm depth, no hyphae were detected.

### Imaging of AMF extraradical structures in the *Hyphosphere* setup

**Fig. 5:**
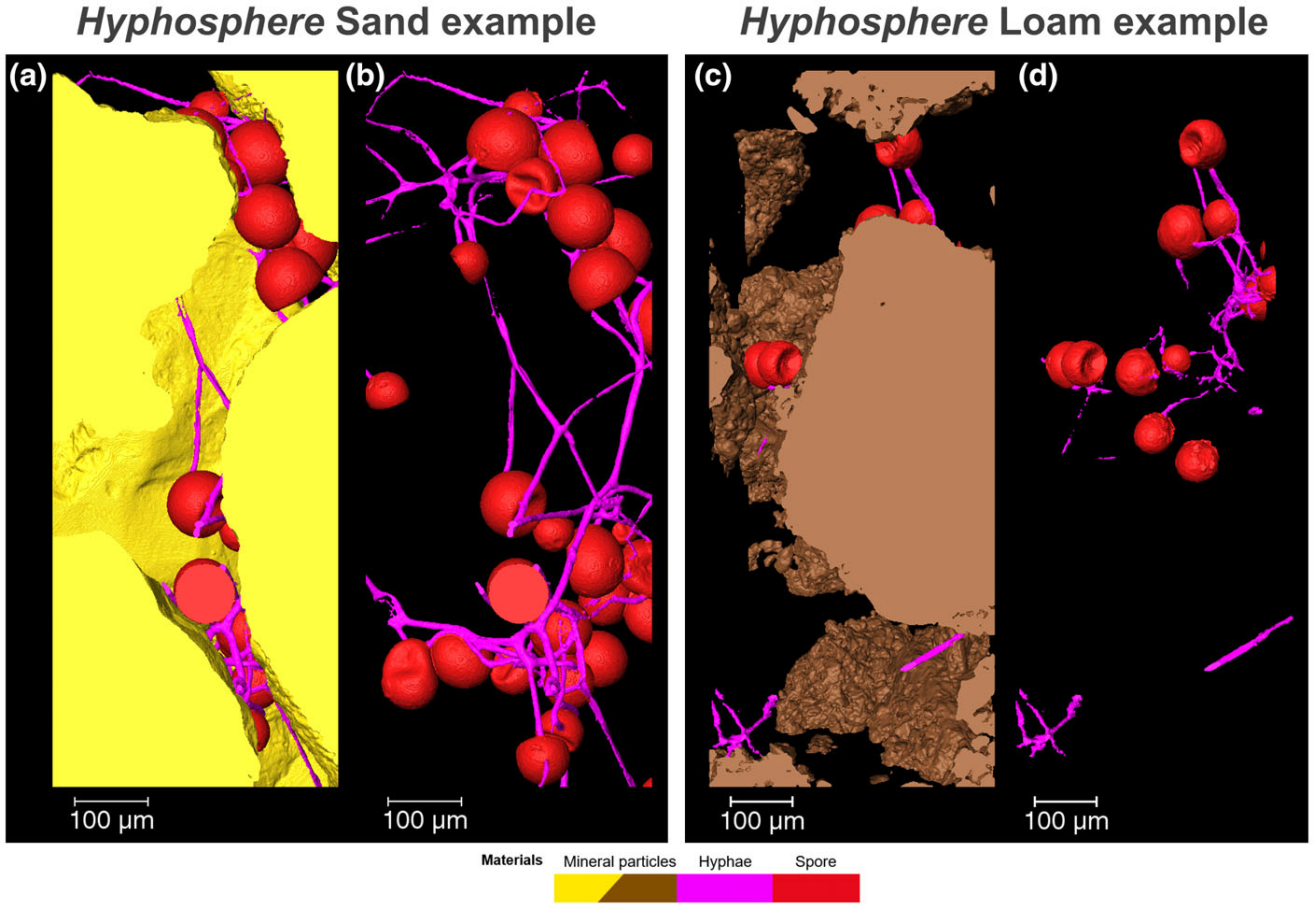
Exemplary three‐dimensional–segmented *Hyphosphere* spore clusters and hyphal networks of an arbuscular mycorrhizal fungus (*Rhizophagus irregularis*) (voxel size: 0.65 μm, dimensions: 975 × 390 × 390 μm) in sand (a, b; yellow) and loam (c, d; brown). In both examples, the same hyphae (purple) and spores (red, round shape) are shown in soil (a, c) and with soil removed (b, d), respectively.

In both soil textures, hyphae displayed comparable diameters (*c*. 6 ± 2 μm) to those observed in the *Sterile Hyphosphere* setup, forming continuous networks that connected soil particles (Figs 5, S2). Additionally, newly formed spore clusters were detected. For *F. mosseae*, spores were repeatedly embedded within soil aggregates and partially or fully surrounded by mineral particles (Fig. S3a). By contrast, *R. irregularis* spores occurred both in intra‐ and interaggregate pore space (Figs 5, S3b, S4) and were also observed once inside a dead root (Fig. S3c).

The contrast between solid structures and the air‐filled pore space provided sufficient differences in gray values to allow segmentation of soil particles, hyphae, and spores based on intensity and morphology (Fig. 2). This approach was effective for both soil textures, although the signal‐to‐noise ratio was higher in the coarse sand than in the silty loam. Across two exemplary 3D volumes, pieces of the AMF hyphal network could be successfully segmented using the previously described workflow. Following the image analysis pipeline (Fig. 3), we quantified geometric and topological features of the segmented AMF structures. The resulting output parameters from the segmented volumes are summarized in Table 1. These values are not intended to represent species‐ or soil‐specific trends; rather, they illustrate the range of geometric parameters that can be extracted using our image analysis workflow. These include counts of AMF objects, total hyphal length and length density, hyphal and spore volume, surface area, hyphal diameter, and volume‐to‐surface‐area ratios. Additional hyphal traits comprise the skeletal branch length and tortuosity. Interface metrics include hypha‐spore, hypha‐soil, and spore‐soil contact areas, from which we derived the proportion of hyphal and spore surface area in direct contact with soil, as well as the fraction of soil particle surfaces covered by AMF structures. These values are likely underestimations because hyphae become harder to detect the more intimately they are associated with soil particles, particularly in the loam. Finally, we quantified the reduction in pore diameter and pore volume resulting from the presence of AMF structures occupying the pore space.

**Table 1:** Selected output parameters of image analysis of *Hyphosphere* example volumes shown in Fig. 5.

Geometric measurement	Sand example		Loam example	
	Hyphae	Spores	Hyphae	Spores
No. of branches/structures	134	32	50	14
Total length (μm)	8280	–	2287	–
Hyphal length density (m cm^−3^)	55.83	–	15.42	–
Total volume (μm^3^)	437 151	6375 555	100 060	1840 552
Total surface area (μm^2^)	233 868	537 282	67 461	185 788
Volume:surface area ratio	1.87	11.87	1.48	9.91
Average local diameter ± SD (μm)	6.18 ± 2.85	57.61 ± 19.80	5.18 ± 1.47	48.41 ± 22.01
Average branch length (μm)	101.0	–	45.7	–
Average tortuosity	1.27	–	1.26	–
Soil contact area (μm^2^)	12 159	13 914	3414	12 513
Soil contact area per total AMF‐structure's surface (μm^2^ μm^−2^)	5.20%	2.59%	5.06%	6.73%
Soil surface covered by AMF‐structure (μm^2^ μm^−2^)	0.57%	0.65%	0.12%	0.43%
	**Hyphae + Spores**		**Hyphae + Spores**	
Hyphae‐spore contact area (μm^2^)	4372		1149	
Average pore size diameter with AMF (AMF structures removed) (μm)	125.2 (139.7)		230.7 (235.5)	
Relative pore size diameter reduction by AMF (μm μm^−1^)	10.4%		2.1%	
Relative pore size volume reduction by AMF (μm^3^ μm^−3^)	28.0%		6.0%	

AMF, arbuscular mycorrhizal fungi. The volumes contain an arbuscular mycorrhizal fungal (AMF, species: *Rhizophagus irregularis*) spore cluster and hyphal network in coarse sand and silty loam, respectively. The volumes analyzed had dimensions of 975 × 390 × 390 μm. Note that these values are not representative for a whole fungal mycelium.

### Imaging of AMF extraradical structures in the *Rhizosphere* setup

**Fig. 6:**
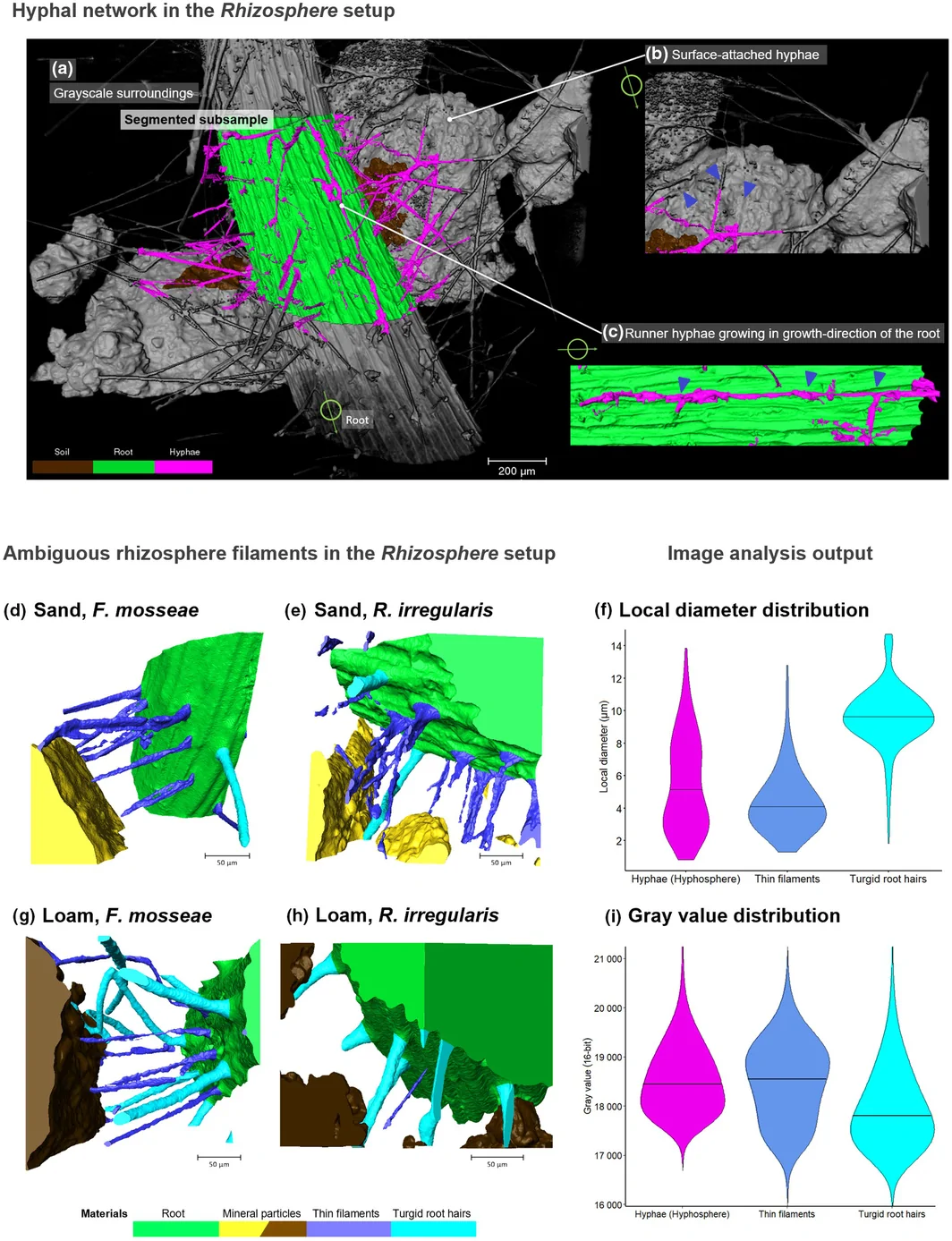
Segmented exemplary *Rhizosphere* volumes of roots colonized by arbuscular mycorrhizal fungi. (a) Three‐dimensional volumetric rendering (partially segmented), of a *Rhizophagus irregularis–colonized Solanum lycopersicum* (cv 76R) root from the *Rhizosphere* setup imaged in loam after 8 wk of growth. In the segmented part, soil (brown), roots (green), and hyphae (purple) are displayed in color. The root surface is densely colonized by *R. irregularis*, with numerous hyphae attaching to and extending from the rhizoplane. Magnified views (b, c) show hyphal attachment (blue arrows) to the soil surface as well as runner hyphae growing along the root surface. (d, e, g, h) Segmented 3D input volumes (edge length: 195 μm) showing mineral particles (sand in yellow, loam in brown color), *Solanum lycopersicum* (cv 76R) roots (green), turgid root hairs (light blue), and thin filaments (blue). The shown ambiguous examples are root sections that contain both thicker and thinner filaments. (f) Violin plot with horizontal median line showing local diameter measurements of root hairs (light blue) and thin filaments (blue) from *Rhizosphere* cores compared to hyphae from the *Hyphosphere* cores (purple, Fig. 5; Table 1) as well as corresponding gray value measurements (i).

**Fig. 7:**
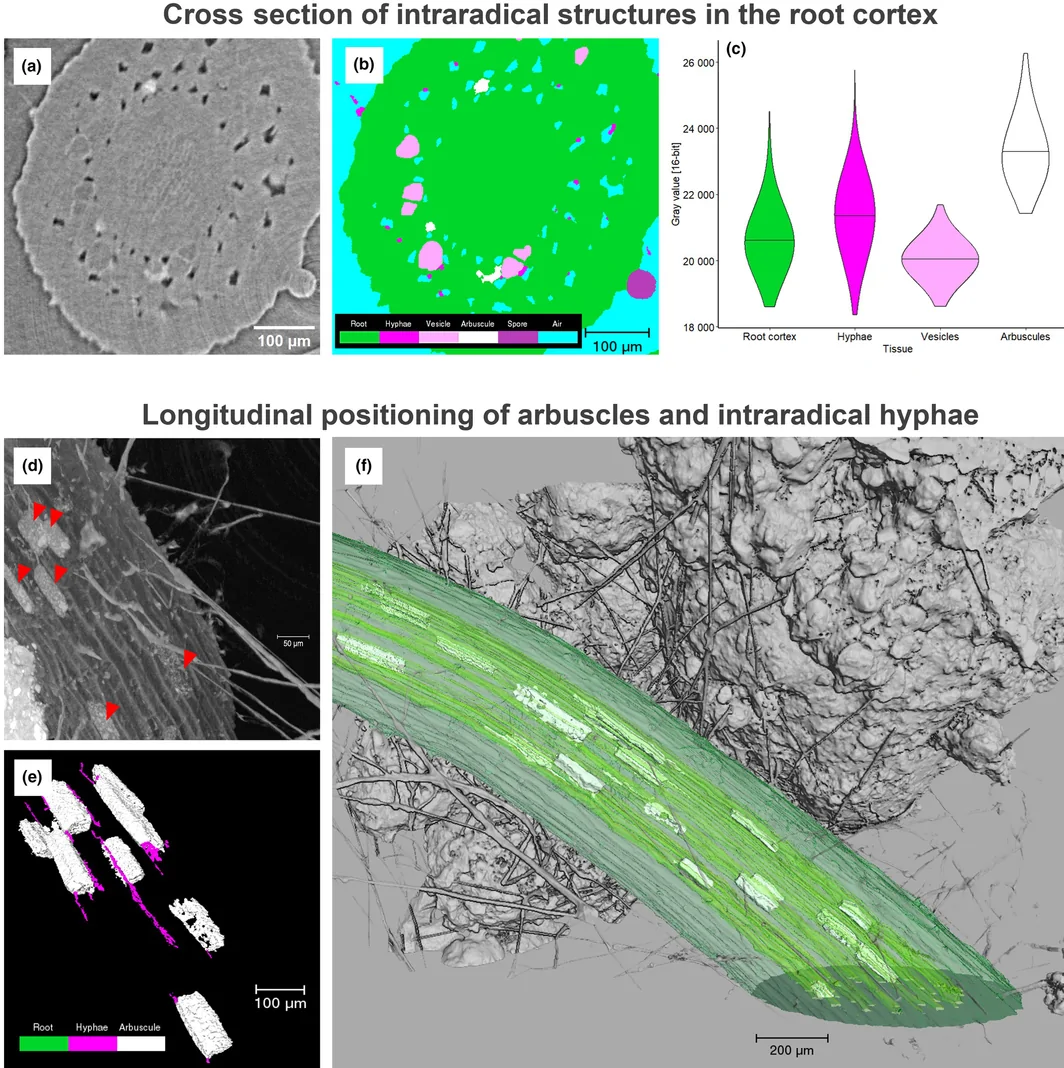
Grayscale images and segmentations of intraradical arbuscular mycorrhizal fungal (AMF) structures in *Rhizophagus irregularis*‐colonized *Solanum lycopersicum* (cv 76R) roots. In the images, segmented materials include roots (in green), hyphae (pink), vesicles (light pink), arbuscules (white), a spore (purple, round shape at the bottom right), and air (light blue). Upper panel, cross section through a colonized root: (a) 2D grayscale slice showing intraradical structures; (b) corresponding 2D‐segmentation; and (c) violin plot with horizontal median line of the gray value distributions of different tissues. Lower panel, longitudinal positioning of AMF structures: (d) Computed microtomography grayscale image showing extraradical hyphae and bright arbuscules (red arrows) in the underlying root cortex; (e) 3D segmentation of arbuscules and intraradical hyphae within the same 600‐μm root segment; (f) same 3D segmented arbuscules in the root cortex in soil (extraradical soil structures have been left gray).

The *Rhizosphere* setup allowed root ingrowth and hence enabled imaging of mycorrhizosphere soil. The overview scans (voxel size: 2.8 or 3 μm, FOV: *c*. 12 × 12 × 6 mm) contained 1–28 individual roots each, regularly showing putative intraradical AMF structures in roots as well as hyphae and spores in the soil pore space. In the high‐resolution scans (voxel size: 0.65 μm), AMF structures could be observed in detail (Fig. 6). In multiple scans, the AMF hyphal network was visibly growing toward and along the root surface and connecting the root with soil particles. The network reached distances of more than 5 mm from the root's surface. In *Rhizosphere* samples, the total number of filamentous structures was higher with more frequent branching compared to root‐excluding ingrowth cores (*Sterile Hyphosphere* and *Hyphosphere* setups). A major difficulty in *Rhizosphere* setups was to distinguish AMF hyphae from root hairs, as both are filamentous structures emerging from the root. We considered structures with low diameter, frequent branching, surface‐attached growing patterns, and root hair untypical connection to the root as AMF hyphae. Following these criteria, a root piece with no root hairs is depicted in a volumetric rendering (Fig. 6a). Here, the majority of AMF hyphae has a diameter below 7 μm, similar to the *Hyphosphere* setup. Most hyphae branched both in mid‐pore space as well as close to soil particles (Fig. 6a,b). Additionally, hyphal connection or entry points on the root surface could be observed either as a single occurrence or as runner hyphae, which grew close to the root surface and connected to it multiple times (Fig. 6c). Another potential proof of local colonization of the depicted root piece is the presence of arbuscules and intraradical hyphae in the underlying root cortex (see the ‘Imaging of intraradical AMF structures in the *Rhizosphere* setup’ section; Fig. 7f).

Next, our goal was to find measures to reliably differentiate AMF hyphae from root hairs in *S. lycopersicum* rhizosphere samples when both structures are present. For the four example volumes shown in Fig. 6(d,e,g,h), we segmented all filamentous structures and quantified their geometrical properties and gray value distributions. We evaluated turgid root hairs and hyphae‐like (thin filament) structures using the same metrics applied previously in *Hyphosphere* analyses (Table 1), including the following: number of connected structures, volume, surface area, internal diameter, volume‐to‐surface area ratio, branch length, tortuosity, soil and root interface areas (Table S4). Additionally, the average root interface area was 1004 μm^2^ for a single turgid root hair and 773 μm^2^ for a thin filament. For these small volumetric snippets, root contact and soil contact areas were of comparable magnitude across both structure classes. We further compared local diameter and gray value distributions of turgid root hairs and thin filaments with those of AMF hyphae previously segmented in *Hyphosphere* samples (Fig. 6f,i). Keeping in mind that diameter is intrinsically part of the classification criteria, our exemplary analysis of local diameter and gray value showed that the thin filaments rather matched AMF hyphae and were distinct from the thicker, lower gray value turgid root hairs.

### Imaging of intraradical AMF structures in the *Rhizosphere* setup

High spatial resolution and phase contrast enabled *in situ* detection of intraradical AMF structures within roots in soil. In this study's micro‐CT images of *S. lycopersicum* roots, three distinct AMF‐like structures were identifiable: (1) Arbuscule‐like structures: the most frequently observed structures were sac‐shaped regions occupying part or all of inner cortical cells. These features exhibited markedly elevated and highly variable gray values (23 352 ± 1509) relative to the surrounding cortex tissue (20 573 ± 668). Their average dimensions were 79 ± 17 μm in length and 26 ± 3 μm in width. Their size, location outside the central cylinder, and the heterogeneous gray value pattern support the interpretation as arbuscules (Fig. 7). Dense networks of fine arbuscular branches could generate such strong phase‐contrast signals. In an example segmented root segment (length 2.0 mm; Fig. 7a), their volumetric contribution to the root cortex was 0.59%. (2) Intraradical hyphae: a second class of structures consisting of narrow, tubular features extending longitudinally between cells within the cortex. These were frequently connected to the sac‐like arbuscule‐filled cells, consistent with intraradical morphology expected for the used AMF species in *S. lycopersicum* roots (Cavagnaro *et al*., 2001). Even at the highest resolution, intraradical hyphae were only partially detectable; however, those that were visible had diameters of 5.7 ± 1.1 μm. Their alignment with the direction of root elongation further supports their identity. (3) Vesicle‐like structures: we observed large (22.0 ± 5.6 μm), round to ellipsoidal features with lower gray values (20 002 ± 624) than the surrounding cortex (Fig. 7b). Their size range, morphology, and lower absorption contrast (lipids are less dense than average root tissue) indicate that these structures are likely vesicles.

To further confirm that the described intraradical structures were indeed AMF structures, we embedded exemplary fresh, *R. irregularis*‐colonized *S. lycopersicum* roots in 1% agarose, and imaged single roots using lab‐based X‐ray microtomography (Fig. S5) and then stained the AMF structures using the ink‐acid method (Vierheilig *et al*., 1998). As with synchrotron‐based micro‐CT, arbuscules and vesicles were visible in both the tomographs and under a light microscope in identical root pieces.

## Discussion

### Setup *Sterile Hyphosphere* for imaging AMF structures in absence of other microbes

We demonstrated that synchrotron‐based micro‐CT imaging allows visualization of AMF structures across increasing levels of biotic complexity. As a first step, we used Petri dish–based root‐organ cultures (the *Sterile Hyphosphere* setup) to image *R. irregularis* hyphae in sterile soil‐filled ingrowth cores, establishing a baseline for recognizing fungal structures in more complex systems. AMF hyphal density inside the ingrowth cores was markedly lower than in the adjacent Phytagel medium, suggesting that *R. irregularis* partially avoided colonizing the ingrowth cores. It is also possible that a fraction of the hyphal network occupied fine pore domains with insufficient contrast for micro‐CT detection. Potential improvements to address this limitation include incorporating compounds known to stimulate AMF foraging (Bukovská *et al*., 2018, 2021) to enhance hyphal exploration of the soil in the ingrowth core. Likewise, the Phytagel medium could be replaced with sterile soil and a nutrient solution, to better mimic natural soil geometry. Although limited to a small range of AMF species, this setup could be a useful platform for studying the three‐dimensional architecture and morphology of the AMF mycelium in soil in a controlled environment. It could be extended to study the mycelium's architecture and morphology in the presence or absence of various non‐filamentous soil organisms such as various protozoa, bacteria, and archaea.

### Setup *Hyphosphere*: imaging of extraradical AMF structures in unsterile soil

We successfully imaged newly formed extraradical mycelia including spore clusters in two contrasting soil textures. This extends earlier work by Keyes *et al*. (2022), who imaged hyphae only in a sandy soil. Their approach had soil‐filled 5‐mm root‐excluding tubes connected at a predefined angle to a plant compartment. By contrast, our ingrowth core‐design allows scanning larger subvolumes (13 mm diameter) and does not impose an angle to hyphal growth. An additional advantage is the flexibility of this system, as ingrowth cores can be incorporated into any pot experiment without requiring specialized growth chambers or designs. The setup was applicable to two different AMF species, namely *R. irregularis* and *F. mosseae* (Table S2), and should readily support imaging of other AMF species or ectomycorrhiza.

Hyphal diameter distributions measured in our *Hyphosphere* sand and loam exemplary volumes, 6.18 ± 2.85 μm in sand vs 5.18 ± 1.47 in loam (Table 1; Fig. 6f) were slightly higher than those reported for destructive analyses (Drew *et al*., 2003; averages of 3.3–4.8 μm, Fig. S6: 4.1 ± 2.3 μm). The total hyphal lengths observed in the *Hyphosphere* setup were of the same order of magnitude as destructive measurements (Drew *et al*., 2003; Keyes *et al*., 2022), although our analyzed examples represent locally dense colonization hotspots. Drew *et al*. (2003) documented a shift toward larger hyphal diameters in sand, a pattern qualitatively reproduced in our exemplary volumes (Fig. 5; Table 1). Average skeletal branch lengths were rather short (Table 1; 46 μm in silty loam, 101 μm in coarse sand), probably due to the examples being highly branched spore clusters and occasional gaps in hyphae due to segmentation errors. Thus, the branch length measure does not reflect the length of actual ‘biological’ branches, as cytoplasmic continuity cannot be assessed from micro‐CT images. Hyphal tortuosity values corresponded well with those measured by Keyes *et al*. (2022), with a mean of 1.26 ± 0.09 across 60 analyzed filaments. Additional traits, including hyphal and spore volumes, surface areas, and surface‐to‐volume ratios, could be extracted from the segmented structures. Micro‐CT therefore provides a means to relate local hyphal and spore morphology to soil microstructure and to investigate how AMF adjust their growth to pore‐scale physical constraints. These data are also suitable for integration into mathematical models.

Mycorrhiza–soil interface areas can now be quantified, which is of particular interest given the important role of AMF in water and nutrient transport. Surprisingly, only *c*. 5% of the total surface area of the analyzed hyphal networks was in visible contact with larger soil particles, despite the widely recognized role of hyphae in entangling soil particles and promoting soil aggregation (Kohler *et al*., 2017). However, it has to be acknowledged that hyphae in intimate contact or surrounded by soil particles were not fully segmented, particularly in loam. Consequently, the present approach primarily quantifies interface areas between hyphae bridging larger pore spaces with sand‐sized particles or soil aggregate surfaces, while interfaces within densely packed soil domains remain challenging to resolve. Spore‐soil contact area was of a similar magnitude compared to that of hyphae, at least within the exemplary volumes containing intentionally selected spore clusters (Fig. 5). These values should not be extrapolated directly to larger soil regions, because the analyzed volumes represent localized hotspots. Bitterlich *et al*. (2018) hypothesized that AMF can modify soil hydraulic connectivity and reduce water‐flow velocities. Our exemplary segmentations (Fig. 5) demonstrate that both hyphae and spores can locally constrict pore pathways. This was quantified as a reduction of local pore diameters and pore volumes of up to −10.4 and −28.0%, respectively, in coarse sand (Table 1). This illustrates that AMF structures can alter pore geometry at the microscale, with potential consequences for water retention, flow pathways, and hydraulic conductivity in soil.

### Setup *Rhizosphere*: imaging of AMF structures in the vicinity of roots in unsterile soil

A major challenge was to differentiate AMF hyphae from shrunken root hairs. We applied a set of morphological criteria derived from known structural characteristics, with diameter and branching as the primary distinguishing features. In the micro‐CT images, individual hyphae could sometimes be traced over several hundred micrometers to determine their growth pattern. Hyphae consistently followed or closely adhered to surfaces – an attribute observed in both micro‐CT datasets (Figs 5, 6) and in light microscopy images (Fig. 1b). Still, these criteria were not always sufficient to unambiguously distinguish between different filament types. Thus, we operationally defined turgid root hairs as unbranched, radially emerging structures with homogeneous diameters exceeding those typical of AMF hyphae (> 6 μm, *Hyphosphere* examples; Drew *et al*., 2003). Under wet conditions, turgid root hairs were readily distinguishable, exhibiting diameters more than twice those of the thin filaments (Table S4). Analysis of stained hyphae under a light microscope yielded diameters of 3.48 ± 2.03 μm (*Hyphosphere*) and 4.94 ± 2.30 μm (*Rhizosphere*), while the unstained root hairs had diameters of 8.19 ± 2.05 μm (Fig. S6). The parameters of the thin filaments detected by micro‐CT, including diameter, branch length, and tortuosity, were generally consistent with hyphae from the *Hyphosphere* setup (Table S4). However, a smaller average diameter (4.33 μm) indicates that some thin filaments might represent shrunken root hairs, whose collapsed morphology strongly reduces their diameter (Duddek *et al*., 2022). In our setups, plants were grown around field capacity, and most root hairs are expected to be turgid, but some uncertainty remains: Soil heterogeneity can generate locally drier or hydraulically disconnected patches, or the root hairs may experience senescence or mechanical damage. Consequently, shrunken root hairs may inadvertently be classified as AMF hyphae. Morphologically, shrunken root hairs exhibit unique features: an ‘emptied fire‐hose’ appearance with a band‐like cross‐section, thicker margins, occasional twisting along the axis, and, importantly, an absence of branching (Duddek *et al*., 2022, supplements). Systematic comparison of these traits with AMF hyphae, potentially using root hair‐defective mutants (Fig. S7), remains an important next step to refine classification in future micro‐CT‐based studies.

While earlier studies have counted physical contact points between AMF hyphae and roots (Hart & Reader, 2005), no previous work has quantified the contact area (interfacial area) in an undisturbed soil environment despite its relevance for water and nutrient transport, carbon allocation, and metabolic and microbiome (biofilm) coupling between symbiotic partners. Even though we cannot yet fully distinguish between hyphae and shrunken root hairs at the root–soil interface, we nonetheless gain new insights into what is possibly one of the most important bottlenecks for plant resource uptake, especially in drying soils (Abdalla *et al*., 2023). In future research, the effects of AMF on root–soil contact and resource gradients could be conservatively evaluated by approximating the filament length across air space in the rhizosphere of AMF‐colonized vs nonmycorrhizal plants.

We showed that micro‐CT can resolve intraradical AMF structures *in situ*, including arbuscules, vesicles, and segments of intraradical hyphae. Conventional microscopy has long provided high‐resolution observations of these structures, but their three‐dimensional detection and quantification is rather recent. For instance, Strock *et al*. (2019) used laser ablation tomography to analyze AMF structures in a maize root segment and calculated that colonized tissue had a volumetric contribution of 4.7%. Using a lab‐based X‐ray microscope and a contrast agent, Duncan *et al*. (2023) showed an example of how intraradical AMF structures can be imaged *in situ*. Our method demonstrates the three‐dimensional detection within an intact soil–root matrix and follow‐up quantification of different intraradical AMF organs, without the need for contrast agents. Micro‐CT thus enables not only the quantification of arbuscules and vesicles by numbers and size but also the spatial analysis of their position relative to the fungus' extraradical architecture and morphology.

### Limitations of using micro‐CT imaging

Despite the advances, the presented setups remain subject to a few constraints inherent to micro‐CT. Foremost, the detectability of biological structures depends on their contrast relative to adjacent materials. AMF structures, especially hyphae, become difficult to segment when surrounded by water, because water attenuates X‐rays similar to living organic tissues. Thus, the soil needs to be drained at least to field capacity so that water is drained from macropores, enabling hyphae to be imaged with sufficient contrast. Even under these conditions, residual water in aggregates and the close association of hyphae with fine mineral particles leaves insufficient contrast to resolve intra‐aggregate hyphae (Keyes *et al*., 2022). This limitation complicates any attempt to quantify the relative contribution of pore‐bridging vs intra‐aggregate hyphae to water and nutrient transport – a key question that remains unresolved. Thus, our segmented results can be viewed as a network of hyphae of higher branching orders, in particular the part of the mycelium that extends outward across pores to explore new areas of the substrate and establish new sites for nutrient uptake.

A second constraint is the trade‐off between resolution and FOV. Detecting AMF hyphae requires high resolution (< 3 μm voxel size). Such scans necessarily restrict the FOV; in our case, the volume of high‐resolution scans was *c*. 80 times smaller than the overview scans. While the latter provided spatial context, their voxel size (2.8–3 μm) was insufficient to segment hyphae reliably. As a result, only a limited fraction of the soil volume can be imaged at a resolution adequate for reconstructing the mycelium. This makes it challenging to obtain representative estimates of hyphal density in heterogeneous soils, given the high spatial variability of AMF structures. Thus, while the method allows unprecedented visualization of AMF structures *in situ*, extrapolations to larger soil domains must be made cautiously. A major bottleneck in micro‐CT is often the segmentation – the images in this study were segmented semi‐automatically with major time effort. Recent developments also allow for automatic deep learning segmentation of complex soil constituents that requires little or no postcorrection (Phalempin *et al*., 2025). Similar algorithms can likely be used to segment different AMF structures in soil.

### Modeling potentials and perspective

Future modeling work could make use of well‐trained segmentation algorithms that allow for automated segmentation of the micro‐CT images. This would enable us to retrieve representative values of three‐dimensional architectural and morphological traits of AMF networks in undisturbed soil. The values can be used to derive important traits, such as the spatial arrangement of pore‐bridging hyphae, their local morphological traits as well as interface areas and connectivity that hyphae provide between roots and soil. Such traits cannot be obtained with any other noninvasive technique to date. For a mechanistic understanding, AM fungal architecture and morphology extracted from micro‐CT images would need to be linked to physical, chemical, and hydrological processes at the root–soil interface. Modeling, informed by such data, will provide an adequate framework for interpreting and scaling up the functional consequences and is an essential tool for translating the microscale observations into system‐scale predictions. A remaining challenge concerns the parametrization of hyphal surface properties and hyphal water transport mechanisms. So far, hydraulic properties such as hyphal conductance or contact angle as a function of matric potential remain unquantified. Such parameters will substantially influence model predictions of the amount of water that a hyphal ‘liquid bridge’ can transport, and at what matric potential AMF hyphae effectively bridge otherwise disconnected pores. While these data gaps remain, micro‐CT images provide realistic *in situ* AMF geometries that could be used for image‐based simulations in the future, thereby allowing us to test how well *in vitro* observations generalize to soil processes. From our micro‐CT images of the ‘soil obstacle courses’ imposed on hyphae, it appeared that the AMF structures had a more patchy distribution in comparison with their more homogeneous growth in Petri dish–based setups (e.g. Oyarte Galvez *et al*., 2025).

Functional‐structural plant models (FSPMs) provide a powerful framework for exploring the dynamic, spatially explicit interactions between root systems, AMF, and soil. Unlike isolated snapshots, FSPMs simulate the codevelopment of roots and fungi in space and time, allowing continuous comprehensive and nondestructive analysis. For example, hyphal surface area and their interface area to roots and soil can affect water and nutrient exchange coefficients, while local hyphal tortuosity can impact effective diffusion tensors (analogous to Schnepf *et al*. (2022) for mucilage bridges). The 3D hyphal architecture parameterized from soil‐grown root systems can also broaden the insights gained from the 2D, whole‐mycelium perspective of Oyarte Galvez *et al*. (2025). This allows for a better understanding of how fungi adapt their growth strategy to the physical constraints of real soil. Future approaches could then couple 3D hyphal geometry with dynamic functions such as carbon allocation, nutrient uptake, and hydrological feedbacks between AMF, roots, and soil. Integrating micro‐CT with complementary imaging techniques such as nanoscale secondary ion mass spectrometry, X‐ray fluorescence, neutron tomography, and destructive microscopy will enable comprehensive model validation and facilitate a mechanistic understanding of AMF foraging and resource exchange in realistic soil environments.

## Competing interests

None declared.

## Author contributions

MB, AS, J Pausch, JJ and MAA acquired the funding. HMB, MB, NK, AS, J Pausch, JJ and MAA designed the study. MB, SD and MAA were lead applicants for the synchrotron sessions at Synchrotron SOLEIL. HMB, MB, NK, EJ, J‐PS, BH, SD, J Perrin, AK, JJ and MAA collected the data. HMB and NK analyzed and interpreted the data with input from MAA, MB and JJ. HMB drafted the manuscript. ASH and AS formulated the modeling perspective. All authors critically revised the publication.

## Disclaimer

The New Phytologist Foundation remains neutral with regard to jurisdictional claims in maps and in any institutional affiliations.

## Supporting information


**Fig. S1** Segmentation pipeline in detail.
**Fig. S2** Segmentation and image analysis from *Sterile Hyphosphere* samples.
**Fig. S3** Micro‐CT images of arbuscular mycorrhizal fungal spores in soil.
**Fig. S4** Micro‐CT images of *Rhizophagus irregularis* filled and empty hyphae as well as spores imaged in a *Hyphosphere* setup in coarse sand.
**Fig. S5** Comparison of micro‐CT images of intraradical arbuscular mycorrhizal fungal structures with light microscopy of the identical root piece.
**Fig. S6** Light microscopic measurements of arbuscular mycorrhizal fungal hyphal and *Solanum lycopersicum* L. root hair diameters.
**Fig. S7** Micro‐CT image of a ‘root‐hairless’ barley (*Hordeum vulgare* L.) root segment illustrating unambiguous hyphal identification.
**Notes S1** Measurement settings at the synchrotron beamlines PSICHÉ and ANATOMIX (Synchrotron SOLEIL, St. Aubin, France).
**Notes S2** Classification criteria distinguishing turgid root hairs vs shrunken root hairs or hyphae.
**Notes S3** Step‐wise image analysis workflow in ImageJ.
**Table S1** Overview of properties of the soils used in this experiment.
**Table S2** Combined results of arbuscular mycorrhizal fungal colonization assessments for *Funneliformis mosseae* and *Rhizophagus irregularis* in our presented setups containing ingrowth cores for micro‐CT imaging (setups *Sterile Hyphosphere*, *Hyphosphere*, and *Rhizosphere*).
**Table S3** Quantitative real‐time polymerase chain reaction assay cycling and primer details.
**Table S4** Selected image analysis outputs for thin filaments and turgid *Solanum lycopersicum* L. root hairs.
Please note: Wiley is not responsible for the content or functionality of any Supporting Information supplied by the authors. Any queries (other than missing material) should be directed to the *New Phytologist* Central Office.

## Data Availability

Data related to this study can be found here, doi: 10.14459/2026mp1856359.
