# Supplementary material for: Opening the black box: in situ imaging of arbuscular mycorrhizal fungal structures in soil using synchrotron‐based micro‐CT
Source: New Phytol. 2026 Jul 2;251(6):3687–702. doi: 10.1111/nph.71379 (PMC13491289; doi:10.1111/nph.71379)
Supplement: Supplementary file 1 [file NPH-251-3687-s001.docx]

***New Phytologist* Supporting Information**

Article title: **Opening the black box: *in-situ* imaging of arbuscular mycorrhizal fungal structures in soil using synchrotron-based micro-CT**

Authors: Henri M. Braunmiller, Michael Bitterlich, Nicolai Koebernick, Eva Jacob, Anna S. Heck, Andrea Schnepf, Johanna Pausch, Jussi-Petteri Suuronen, Bernhard Hesse, Sylvain Delzon, Jonathan Perrin, Andrew King, Jan Jansa, Mutez A. Ahmed

**Article acceptance date: 3 June 2026**

The following Supporting Information is available for this article:

**Table S1.** Overview of properties of the soils used in this experiment.

| **Soil properties** | **Silty loam** | **Coarse sand** |
| --- | --- | --- |
| Bulk density [g cm^-3^] | 1.22 | 1.50 |
| Soil texture [w w^-1^] | 33% Sand  48% Silt  19% Clay | 100% Sand |
| Saturated hydraulic conductivity [cm d^-1^] | 47.9 | 3136 |
| Organic matter  [w w^-1^] | 0.84% | not detected |

w, weight unit.

**Notes S1.** Measurement settings at the synchrotron beamlines PSICHÉ and ANATOMIX (Synchrotron SOLEIL, St. Aubin, France).

At the PSICHÉ beamline, measurements were made using a monochromatic beam at 33 keV with around 3 x 10^11^ photons mm^-2^ s^-1^. The detector system consisted of a Hamamatsu ORCA Flash 4.0 sCMOS camera (Hamamatsu Photonics K.K., Nakaku, Japan). For the overview scans, the system was coupled to a 250 µm thick Lutetium Aluminium Garnet (LuAG) scintillator using a 2.3x objective consisting of two Hasselblad medium format objectives in tandem configuration, resulting in an effective pixel size of 2.8 µm. X-ray projections were recorded using 18 ms exposure time, with either 1500 projections over 180 degrees or 5900 projections over 360 degrees with an offset rotation axis. Overview scan reconstructions were used to identify regions of interest prior to switching to high resolution mode. The high-resolution detector system consisted of a Hamamatsu ORCA Flash4.0 sCMOS camera, coupled to a 50 µm thick LuAG scintillator using a 10x microscope objective, resulting in an effective pixel size of 0.64 µm. X-ray projections were recorded using 300 ms exposure time during continuous rotation of the sample. 1500 projections were recorded during a rotation of 180 degrees.

At the ANATOMIX beamline, the scanning was done utilizing a filtered white beam with approximately 45 keV mean energy. The projection images (3000 images over a full 360 degrees rotation) were recorded with a Hamamatsu Orca Flash 4.0 camera (Hamamatsu Photonics K.K., Nakaku, Japan) coupled to a LuAG scintillator, of 300 µm thickness for the overview scans, 20 µm thickness for the high-resolution scans. Exposure time per projection was 35 ms for the overview scans, 150 ms for high-resolution scans. A small marker formed of aluminum copper wires was taped on the exterior of each hyphal compartment before scanning, and used as a landmark to assist in positioning the intended ROI in the FOV of the high-resolution scan. For the high-resolution scanning, the center of rotation of the scan was offset to near to the edge of the detector, to extend the horizontal field-of-view of the scan to 2.6 mm.

The data were reconstructed using PyHST2 (Mirone *et al.*, 2014; King *et al.*, 2016), after applying a phase retrieval filter (Paganin *et al.*, 2002), and stored as 16-bit grayscale.raw files.

**Fig. S1** Segmentation pipeline in detail. By using thresholding, fill holes for dense and intermediate density structures, arbuscular mycorrhizal fungal (AMF, species: *Rhizophagus irregularis*) structures and minerals can be distinguished. With manual assignment and correction, we get a final output image with a mineral (yellow), a hyphal (purple), a spore (red) and an air phase (light blue).

**Notes S2.** Classification criteria distinguishing turgid root hairs vs. shrunken root hairs or hyphae.

(1) Diameter: Turgid tomato root hairs can exceed 20 µm in diameter (Cormack, 1945), while the majority of AMF hyphae fall below 8 µm (Drew *et al.*, 2003). We analyzed turgid tomato root hairs and *R. irregularis* hyphae with light microscopy and found that most hyphae fall below 6 µm, whereas root hairs exceeded this diameter ( SI Fig. S9). Only some hyphal types - runner hyphae or germ tubes - occasionally approach 15-20 µm (Friese *et al.*, 1991). (2) Branching frequency: Turgid root hairs typically have larger, uniform diameters and no branching, while hyphae characteristically branch, often extensively near roots as reported previously for *F. mosseae* (Giovannetti *et al.*, 2001). (3) Surface-attached growth patterns: We assume that turgid root hairs either penetrate loose aggregated fine particles (Koebernick *et al.*, 2017) or are bent by hard surfaces (sand grains or dry aggregates) rather than closely adhering to them ( Duddek *et al.*, 2022) and observed in the present study. (4) Connection to the root surface: Root hairs emerge cylindrically from individual epidermal cells, each forming from a rounded protuberance and extending from a bulged ridge (“papilla”, Cormack, 1945). In contrast, AMF structures form hyphopodia or surface-attached hyphae.

**Notes S3:** Step-wise image analysis workflow in ImageJ.

The described workflow (Fig. 3 main document) can be completed in *c.* 1.5-2 hours (for a volume of 600 x 600 x 1500 voxels) using a professional working station (CPU: Intel® Xeon® 5418Y, Intel Corporation, Santa Clara, USA, at 2.00 GHz base speed, 3.80 GHz maximum speed, with 48 cores). The most time consuming steps being the skeletonization and the creation of local thickness maps in the pore space (*c.* 5 min and 30 min respectively).

*1. Morphology (with MorphoLibJ extension,* Legland *et al.*, 2016*)*

The 3D images segmented in Avizo then only carry gray values of n+1 the number of materials n. Each material is assigned a unique gray value (e.g. soil=1, hyphae=2, spore=3 and so on). The first step is then to threshold for the material, convert it to a mask and to run a 3D Median filter to smooth the surface.

Code for hyphae (value=2) as an example:

setThreshold(2, 2);

setOption("BlackBackground", false);

run("Convert to Mask", "background=Dark");

run("3D Fast Filters","filter=Median radius_x_pix=2.0 radius_y_pix=2.0 radius_z_pix=2.0 Nb_cpus=48");

Then, to detect individual, connected regions:

run("Find Connected Regions", "allow_diagonal display_one_image display_results regions_must regions_for_values_over=100 minimum_number_of_points=500 stop_after=-1");

**Fig. Notes S3-1** Exemplary arbuscular mycorrhizal fungal (AMF, species: *Rhizophagus irregularis*) hyphal network from Fig. 5 and its image analysis processing steps. From left to right: hyphal network including spores (hyphae in purple, spores in red); only hyphae; connected structures and finally, the skeletonized hyphae.

And follow-up quantification:

run("Analyze Regions 3D", "volume surface_area mean_breadth euler_number surface_area_method=[Crofton (13 dirs.)] euler_connectivity=26");

**Output: Number of structure, volume, surface area, mean breadth and euler number**

For the interface area between two materials (e.g. hyphae with value 2 and spore with value 3):

run("Interface Surface Area", "label=3 label_0=2 surface_area_method=[Crofton (13 dirs.)]");

**Output: contact area of hyphae and spores to soil or root**

*2. Skeleton morphology (with BoneJ extension,* Domander *et al.*, 2021*)*

run("8-bit");

run("Skeletonize (2D/3D)");

And then the skeleton analysis:

run("Analyze Skeleton (2D/3D)", "prune=none calculate show display");

**Output: branch length, total length (as sum of branch lengths > 40 µm), tortuosity (as branch length divided by euclidean distance)**

*3. Local diameter measurements (with BoneJ extension,* Domander *et al.*, 2021*)*

For that, we run the “local thickness (complete process)” with the phase of interest in the background. Thus, the thresholded stack has to be inverted.

run("Invert", "stack");

run("Local Thickness(complete process)", "threshold=1");

**Fig. Notes S3-2** Local diameter display based on the explained calculations (in spectral colors, white: thick diameters, purple: thin structures). Examples are from Fig. 5 in the main document.

*4. Gray value profile*

Open gray scale image and labeled image (called “gray” and “hyphae label” in the following). The “hyphal label” image is gained from the 3D segmented image and then thresholded and converted to a mask, just like in *1. Morphology*. The tresholded label image is processed, so a value of 1 for hyphal structures remains and every other material is set to a gray value of 0. The image is then multiplied with the gray scale image, resulting in a gray scale image of only the hyphae.

// manually click on labeled image

rename(“hyphae label”);

setThreshold(2, 2);

setOption("BlackBackground", false);

run("Convert to Mask", "background=Dark");

run("Divide...", "value=255 stack");

imageCalculator("Multiply create 16-bit stack","gray","hairs-1");

Then, for the resulting gray value distribution, we run a histogram:

run(“Histogram”);

**Table S2.** Combined results of arbuscular mycorrhizal fungal (AMF) colonization assessments for *Funneliformis mosseae* and *Rhizophagus irregularis* in our presented setups containing ingrowth cores for micro-CT imaging (setups *Sterile Hyphosphere*, *Hyphosphere* and *Rhizosphere*).To verify the establishment of mycorrhizal colonization in the *Hyphosphere* and *Rhizosphere* setup, subsamples of roots from the soil surrounding the ingrowth cores were stained following the acid-ink method of Vierheilig *et al.* (1998). In short, from every root system, 10 positions each in 15 representative 2-cm root pieces were scored for root colonization by AMF (yes/no), resulting in a total of 150 positions per root system. This was done with a light microscope, at a magnification of 200x and a field of view of 1.0 x 1.0 mm for one position. The percentage of colonized vs. total amount of positions then gives the root colonization frequency. A verification of successful colonization of ingrowth cores with the respective AMF species (*R. irregularis* or *F. mosseae*) molecular markers was then performed using quantitative PCR (qPCR) as described previously by Thonar *et al.* (2012). From inside the *Hyphosphere* and *Rhizosphere* ingrowth core, soil and roots with adhering rhizosheaths were analyzed, spiked with taxon-specific molecular markers following Thonar *et al.* (2012), including with an internal DNA standard to evaluate DNA extraction efficiency (SI Table S3).

Hyphal length density ranges (minimum and maximum value as well as mean value in brackets) in *Rhizosphere* soil were additionally measured via extraction, ink staining, and line-intersection microscopy (Bardgett, 1991).

| Assessment method | *Sterile Hypho- sphere* cores  (n = 6) | *Hyphosphere* cores  (n = 12) | *Rhizosphere* cores  (n = 12) |
| --- | --- | --- | --- |
| Optical microscopy | Qualitatively confirmed  (*R. irregularis*) | Root colonization frequency (core-surrounding soil):  • *F. mosseae*: 85-100% (Mean: 94%)  • *R. irregularis*: 86-100% (Mean: 98%) | |
| qPCR  (28S rDNA copies of the target taxon mg^-1^ material; for details refer to Thonar *et al.*, 2012) | — | • *F. mosseae*: 400-5600 (Mean: 2000)  • *R. irregularis*: 1200-23400 (Mean: 10300) | • *F. mosseae*: Soil: 700-2500 (Mean: 1500), Root + Rhizosheath: 600-2700 (Mean: 1500)  • *R. irregularis*: Soil: 4500-13000 (Mean: 10000), Root + Rhizosheath: 3500–84200 (Mean: 37800) |
| AMF Hyphal length density ranges  (m g^-1^ soil; after Bardgett, 1991) | — | — | • *F. mosseae*: 0.10-2.13 (Mean: 0.86)  • *R. irregularis*: 0.17-2.24 (Mean: 1.06) |

AMF, arbuscular mycorrhizal fungi; qPCR, quantitative real-time polymerase chain reaction assay.

**Table S3.** Quantitative real-time polymerase chain reaction assay (qPCR) cycling and primer details. The assays were done using Luna TaqMan master mix (NEB) and a Lightcycler 480 Instrument II (Roche); further details can be found in Thonar *et al.* (2012).

| **Name of the marker** | **Target** | **Amplicon length (bp)** | **Forward primer sequence (5´-3´)** | **Reverse primer sequence (5´-3´)** |
| --- | --- | --- | --- | --- |
| ISC | internal DNA standard | 87 | CGAACCTGGACTGTTATGATG | AATAAACAATCCCCTGTATTTCAC |
| intra | R. irregularis | 250 | TTCGGGTAATCAGCCTTTCG | TCAGAGATCAGACAGGTAGCC |
| moss | F. mosseae | 122 | GGAAACGATTGAAGTCAGTCATACCAA | CGAAAAAGTACACCAAGAGATCCCAAT |

(continuation 1)

| **TaqMan probe sequence (5´-3´)** | **Concentration of forward primer in the reaction mix (nM)** | **Concentration of reverse primer in the reaction mix (nM)** | **Concentration of TaqMan probe in the reaction mix (nM) - if applicable** | **Master mix** |
| --- | --- | --- | --- | --- |
| FAM-CACCAGGCACCAACAACGACCATT-BHQ1 | 500 | 500 | 125 | Luna Universal Probe qPCR master mix (NEB) |
| TTAACCAACCACACGGGCAAGTACA | 500 | 500 | 125 | Luna Universal Probe qPCR master mix (NEB) |
| AGAGTTTCAAAGCCTTCGGATTCGC | 500 | 500 | 125 | Luna Universal Probe qPCR master mix (NEB) |

(continuation 2)

| **Instrument** | **Reaction volume (ul)** | **Template volume (ul)** | **Cycling program: initial denaturation temperature/duration (min), rate of change(°C/min/°C per s)** | **Number of cycles** | **Denaturation temperature(°C)/duration (s)/rate of change (°C/s)** | **Annealing temperature(°C)/duration (s)/rate of change (°C/s)** | **Amplification temperature(°C)/duration (s)/rate of change (°C/s)** |
| --- | --- | --- | --- | --- | --- | --- | --- |
| LightCycler 480 II (Roche) | 20 | 2 | 95/5/4.4 | 50 | 95/10/4.4 | 54/60/2 | 72/25/4.4 |
| LightCycler 480 II (Roche) | 20 | 2 | 95/5/4.4 | 55 | 95/10/4.4 | 52/60/2 | 72/25/4.4 |
| LightCycler 480 II (Roche) | 20 | 2 | 95/5/4.4 | 55 | 95/10/4.4 | 54/50/2 | 72/25/4.4 |

(continuation 3)

| **Final incubation (temperature(°C)/duration (s)/rate of change (°C/s)** | **Conversion of Cp value to copy numbers per ul template** | **Note** | **qPCR efficiency (%)** | **Target concentration range used for calibration (copies per ul template)** | **R^2^** | **Cp value (negative control)** |
| --- | --- | --- | --- | --- | --- | --- |
| 40/1/2.2 | (15458512772225,2*EXP(-0,58*Cp))/2 | FAM - fluorescein, BHQ1 - blackhole quencher | 99.3 | 100-1000000000 | 0.99 | 34.6 |
| 40/1/2.2 | (70807814816,7202*EXP(-0,60454*Cp))/2 | FAM - fluorescein, BHQ1 - blackhole quencher | 83.8 | 81 - 1084877993 | 0.988 | none |
| 40/1/2.2 | (163768238694,197*EXP(-0,56767*Cp))/2 | FAM - fluorescein, BHQ1 - blackhole quencher | 77.4 | 36 - 1782163076 | 0.999 | none |

| **(d)** | **Example volumes** | Total length [µm] | Hyphal length  density [m cm^-3^] | Average diameter ± standard deviation  [µm] | Tortuosity | Soil contact  area [µm] | Relative soil contact |
| --- | --- | --- | --- | --- | --- | --- | --- |
|  | (a) | 2343.2 | 9.12 | 6.02 ± 2.35 | 1.21 | 13138.7 | 13.92% |
|  | (b) | 781.5 | 13.31 | 10.42 ± 2.00 | 1.18 | 2771.8 | 5.57% |
|  | (c) | 165.8 | 15.69 | 6.25 ± 1.22 | 1.18 | 240.0 | 5.99% |

**Fig. S2** Segmentation and image analysis from *Sterile Hyphosphere* samples. (a-c) Segmented *R. irregularis* hyphae (pink color) in the soil matrix (loam, in brown color). (d) On such exemplary segmented volumes, image analysis can be analogously performed to Fig. 4, Table 1 (publication’s main document).

**Fig. S3** Micro-CT images of arbuscular mycorrhizal fungal spores in soil. (a) Micro-CT image of *Funneliformis mosseae* spores (red arrows) inside a soil aggregate and in close proximity to a root (voxel size: 2.8 µm, field of view: 1.0 x 1.0 mm); (b) Micro-CT image of a *Rhizophagus irregularis* spore cluster inside a soil aggregate (voxel size: 2.8 µm, field of view: 0.6 x 0.6 mm) (c) high-resolution micro-CT image of *R. irregularis* spores in a dead root (red arrows; voxel size: 0.65 µm, field of view: 1.25 x 1.25 mm).

**Fig. S4** micro-CT images of *Rhizophagus irregularis* filled and empty hyphae as well as spores imaged in a *Hyphosphere* setup in coarse sand. The empty red arrows point towards empty hyphae, the full red arrows towards setpa in empty hyphae. (a) single slice; (b) 13 µm depth in z-direction.

**Table S4.** Selected image-analysis outputs for thin filaments and turgid *Solanum lycopersicum* L. root hairs. The data was extracted from the segmented examples shown in Fig. 7 (*Rhizosphere* ingrowth cores). Geometric parameters are pooled across all four volumes.

| Geometric measurement | Thin filaments | Turgid root hairs |
| --- | --- | --- |
| Number structures | 35 | 16 |
| Total length [µm] | 2654 | 1748 |
| Length density [m cm^-^³] | 19.68 | 12.95 |
| Total volume [µm³] | 125237 | 150446 |
| Total surface area [µm²] | 90671 | 43243 |
| Volume:surface area ratio | 1.38 | 2.40 |
| Average local diameter [µm] | 4.33 | 9.66 |
| Average branch length [µm] | 44.23 | (83.22) |
| Average tortuosity | 1.23 | 1.19 |
| Soil interface area [µm²] | 25299.7 | 16711.3 |
| Root interface area [µm²] | 19325.6 | 16071.3 |
| Root interface area per filament  [µm²] | 773.0 | 1004.4 |

**Fig. S5** Comparison of micro-CT images of intraradical arbuscular mycorrhizal fungal (AMF) structures with light-microscopy of the identical root piece. (a) Lab-based micro-CT image of intraradical structures with a high gray value. The sack-shaped structures are presumably AMF arbuscules (*Rhizophagus irregularis*) in a *Solanum lycopersicum* L. (cv. 76R) root. (b-c) Corresponding light microscopic image of stained AMF-structures in the identical root piece. Notice that the staining method requires heat and chemical treatment (KOH), thereby destabilizing the root’s shape. Method: A few selected harvested roots were embedded in agarose medium (1%) in an Eppendorf test tube and CT scanned with a TESCAN Unitom HR system (TESCAN, Brno, Czech Republic). Samples were scanned in microfocus mode at 60 kV and 5 W. We recorded 4389 projections using 6 averages at 150 ms exposure time and a spatial resolution of *c.* 3.4 µm voxel side length. Scans were reconstructed using TESCAN’s Panthera software and stored as 16-bit tiff-stacks. After lab-based micro-CT imaging, roots were stained following the acid-ink staining method of Vierheilig *et al.* (1998) and later imaged with 200x magnification with a stereomicroscope.

**Fig. S6** Light microscopic measurements of arbuscular mycorrhizal fungal (AMF) hyphal and root hair diameters. (a) Light microscopy of a stained, AMF structure-free *Solanum lycopersicum* L. root fragment (b) Microscopy of a stained, AMF-colonized root fragment from the *Solanum lycopersicum* L. cv. 76R, showing both internal and external colonization by *R. irregularis*. (c) Extraradical mycelium of *R. irregularis* extracted from a root-free *Hyphosphere* ingrowth core. Projected diameter measurements of root hairs and hyphae are indicated in panels a-c. (d) Violin plots showing projected diameters of hyphae extracted from bulk soil in the root-free compartments, from hyphae at the root surface, and from root hairs of the 76R cultivar. Most fungal hyphae had diameters below 6 µm: hyphae from *Hyphosphere* samples ranged from 0.78 to 13.77 µm (mean: 3.48 ± 2.03 µm), hyphae from Rhizosphere samples ranged from 1.44 to 11.42 µm (mean: 4.94 ± 2.30 µm), while root hairs had diameters ranged from 4.32 to 14.59 µm (mean: 8.19 ± 2.05 µm), never below 4 µm (gray horizontal line).

**Fig. S7** Micro-CT image of a “root-hairless” barley (NRH, *Hordeum vulgare* L.) root segment illustrating unambiguous hyphal identification (0.65 µm voxel size; field of view: 650 × 910 µm). The root is growing in the coarse sand and colonized by *Rhizophagus irregularis*, both detailed in this study. Dense soil particles adhere to the root’s surface. A hyphae and spore are seen on the bottom right (red arrows, bottom right), while two root hair stumps can be seen on the top left (light blue arrows, top left). The NRH barley line was described in Brown *et al.* (2012) and was selected from the barley mutant population at The James Hutton Institute (Caldwell *et al.*, 2004).

**References**

**Bardgett RD. 1991.** The use of the membrane filter technique for comparative measurements of hyphal lengths in different grassland sites. *Agriculture, Ecosystems & Environment* **34**: 115-119.

**Brown LK, George TS, Thompson JA, Wright G, Lyon J, Dupuy L, Hubbard SF, White PJ. 2012.** What are the implications of variation in root hair length on tolerance to phosphorus deficiency in combination with water stress in barley (*Hordeum vulgare*)? *Annals of Botany* **110**: 319-328.

**Caldwell DG, McCallum N, Shaw P, Muehlbauer GJ, Marshall DF, Waugh R. 2004.** A structured mutant population for forward and reverse genetics in barley (*Hordeum vulgare* L.). *Plant Journal* **40**: 143-150.

**Cormack** **RGH. 1945.** Cell elongation and the development of root hairs in tomato roots. *American Journal of Botany* **32**: 490-496.

**Domander** **R, Felder** **AA, Doube** **M. 2021.** BoneJ2 - refactoring established research software. *Wellcome Open Research* **6**: 37.

**Drew EA, Murray RS, Smith SE, Jakobsen I. 2003.** Beyond the rhizosphere: growth and function of arbuscular mycorrhizal external hyphae in sands of varying pore sizes. *Plant and Soil* **251**: 105-114.

**Duddek** **P, Carminati** **A, Koebernick** **N, Ohmann** **L, Lovric** **G, Delzon** **S, Rodriguez-Dominguez** **CM, King** **A, Ahmed** **MA. 2022.** The impact of drought-induced root and root hair shrinkage on root-soil contact. *Plant Physiology* **189**: 1232-1236.

**Friese CF, Allen, MF. 1991.** The spread of VA mycorrhizal fungal hyphae in the soil: inoculum types and external hyphal architecture. *Mycologia* **83**: 409-418.

**Giovannetti** **M, Fortuna** **P, Citernesi** **AS, Morini** **S, Nuti** **MP. 2001.** The occurrence of anastomosis formation and nuclear exchange in intact arbuscular mycorrhizal networks. *New Phytologist* **151**: 717-724.

**King** **A, Guignot** **N, Zerbino** **P, Boulard** **E, Desjardins** **K, Bordessoule** **M, Leclerq** **N, Le** **S, Renaud** **G, Cerato** **M. 2016.** Tomography and imaging at the PSICHE beam line of the SOLEIL synchrotron. *The Review of Scientific Instruments* **87**: 93704.

**Koebernick** **N, Daly** **KR, Keyes** **SD, George** **TS, Brown** **LK, Raffan** **A, Cooper** **LJ, Naveed** **M, Bengough** **AG, Sinclair** **I. 2017.** High-resolution synchrotron imaging shows that root hairs influence rhizosphere soil structure formation. *New Phytologist* **216**: 124-135.

**Legland** **D, Arganda-Carreras** **I, Andrey** **P. 2016.** MorphoLibJ: integrated library and plugins for mathematical morphology with ImageJ. *Bioinformatics (Oxford, England)* **32**: 3532-3534.

**Mirone** **A, Brun** **E, Gouillart** **E, Tafforeau** **P, Kieffer** **J. 2014.** The PyHST2 hybrid distributed code for high speed tomographic reconstruction with iterative reconstruction and a priori knowledge capabilities. *Nuclear Instruments and Methods in Physics Research Section B: Beam Interactions with Materials and Atoms* **324**: 41-48.

**Paganin** **D, Mayo** **SC, Gureyev** **TE, Miller** **PR, Wilkins** **SW. 2002.** Simultaneous phase and amplitude extraction from a single defocused image of a homogeneous object. *Journal of Microscopy* **206**: 33-40.

**Thonar** **C, Erb** **A, Jansa** **J. 2012.** Real-time PCR to quantify composition of arbuscular mycorrhizal fungal communities-marker design, verification, calibration and field validation. *Molecular Ecology Resources* **12**: 219-232.

**Vierheilig** **H, Coughlan** **AP, Wyss** **U, Piche** **Y. 1998.** Ink and vinegar, a simple staining technique for arbuscular-mycorrhizal fungi. *Applied and Environmental Microbiology* **64**: 5004-5007.
